# Geometrically constrained square pyramidal phosphoranide†

**DOI:** 10.1039/d2sc01060g

**Published:** 2022-04-27

**Authors:** Solomon Volodarsky, Irina Malahov, Deependra Bawari, Mohand Diab, Naveen Malik, Boris Tumanskii, Roman Dobrovetsky

**Affiliations:** School of Chemistry, Raymond and Beverly Sackler Faculty of Exact Sciences, Tel Aviv University Department Tel Aviv 69978 Israel rdobrove@tau.ac.il; Department of Molecular Chemistry and Materials Science, Weizmann Institute of Science Rehovot 7610001 Israel

## Abstract

Geometrical constriction of main group elements leading to a change in the reactivity of these main group centers has recently become an important tool in main group chemistry. A lot of focus on using this modern method is dedicated to group 15 elements and especially to phosphorus. In this work, we present the synthesis, isolation and preliminary reactivity study of the geometrically constrained, square pyramidal (SP) phosphoranide anion (1^−^). Unlike, trigonal bipyramidal (TBP) phosphoranides that were shown to react as nucleophiles while their redox chemistry was not reported, 1^−^ reacts both as a nucleophile and reductant. The chemical oxidation of 1^−^ leads to a P–P dimer (1-1) that is formed *via* the dimerization of unstable SP phosphoranyl radical (1˙), an unprecedented decay pathway for phosphoranyl radicals. Reaction of 1^−^ with benzophenone leads *via* a single electron transfer (SET) to 1-OK and corresponding tetraphenyl epoxide (4).

## Abstract 2

Geometrically constrained, square pyramidal phosphoranide was synthesized, and its reactivity study showed that it is both a nucleophile and reductant.

## Graphical abstract

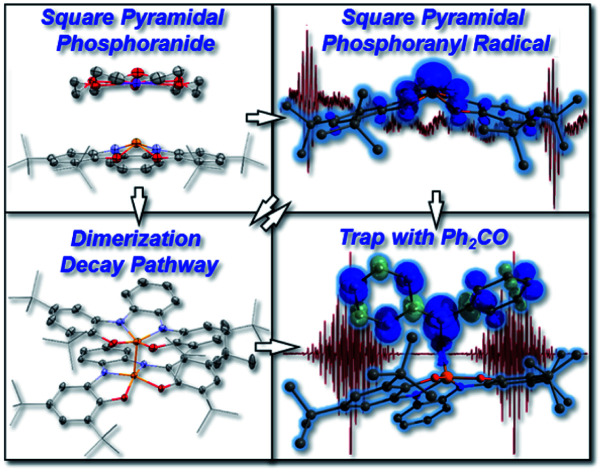

## Introduction

Geometrical constriction of p-block element-based compounds from their VSEPR geometries is emerging as a potent tool to modify their reactivity.^1*a–g*^ Such alterations in geometry are achieved by employing ligands with suitable steric and electronic properties.^1*a–g*^ This approach has recently drawn a lot of attention. For example, Greb recently reported the synthesis and reactivity of a square pyramidal (SP) hydrosilicate, which was surprisingly inert in contrast to its trigonal bipyramidal (TBP) congeners,^2^ abstraction of Cl^−^ from the SP chlorosilicate gave a square planar silane.^3^ The same group later showed the synthesis and reactivity of a planar aluminate that remains Lewis acidic despite the negative charge.^4^ Berionni reported a borane with a pyramidal boron-center having an enhanced Lewis acidity.^5^

A lot of activity in this field is focused on group 15 elements and especially on phosphorus. Embedding the phosphorus atom within a rigid pincer ligand brings about a reorganization of the molecular orbitals that results in a diminished energetic gap between the HOMO and LUMO, rendering the phosphorus center ambiphilic (both nucleophilic and electrophilic). These geometrically constrained phosphines are capable of cycling between stable P^III^/P^V^ oxidation states *via* oxidative addition and reductive elimination type reactions, resembling in a way the chemistry of transition metal complexes. It is important to note, however, that the mechanisms of oxidative addition and reductive elimination in transition metals are completely different. Radosevich reported that planar P^III^ compounds can activate O–H, N–H and C–F bonds at phosphorus center,^6*a–d*^ as well as can act as a catalyst in a hydrogen transfer reaction from H_3_NBH_3_ to azobenzene.^6*d*^ Aldridge and Goicoechea reported a geometrically constrained P^III^ species that could activate E–H bonds in NH_3_ and H_2_O.^7^ We recently reported the synthesis of the first geometrically constrained phosphenium cation, which was able to activate both O–H and N–H bonds, remarkably the activation of the latter was reversible.^8^ Heavier geometrically constrained group 15 analogues such as arsenic, antimony, and bismuth were also synthesized and studied.^9*a–h*^

**Fig. 1:**
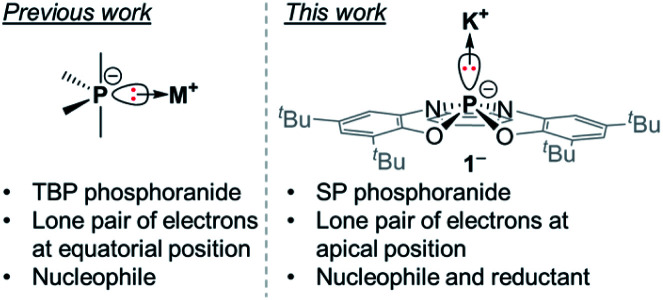
Comparison between TBP phosphoranides and 1^−^.

In our continuous efforts to study the effects of geometrical distortions at phosphorus centers of different P-based ions, we became interested in the class of compounds defined as phosphoranide anions or phosphoranides. Phosphoranides are hypervalent anionic P^III^ species and can be regarded as the conjugate bases of phosphoranes containing an acidic P–H moiety.^10*a,b*^ Phosphoranides were proposed as reactive intermediates (or transition states) in nucleophilic substitution reactions at P^III^ centers prior to their synthesis and isolation.^10*c*^ Despite the fact that P-centres in phosphoranides are four-coordinate, if no metal is directly attached to them, which implies the use of tetrahedral, seesaw, and square planar terminology to describe their geometry, historically however, the accepted terminology to describe these species is as of five-coordinate molecules. This is probably a result of the sterically active lone pair at P-centre that is regarded as a phantom ligand. Thus, similarly to phosphoranes, all of the isolated phosphoranides have a TBP geometry with the lone pair of electrons occupying the equatorial position (Fig. 1), as expected from VSEPR considerations.^11^ Phosphoranides reactivity studies showed that they react as typical P-based nucleophiles, while redox chemistry of these compounds was not explored.^10*a–j*^

We report here the synthesis of the geometrically constrained square pyramidal (SP) phosphoranide (1^−^), which to the best of our knowledge is the first structurally characterized phosphoranide in a SP geometry (Fig. 1). Preliminary reactivity studies showed that 1^−^ is not only nucleophilic, but can also participate in redox reactions.

## Results and discussion

**Scheme 1:**
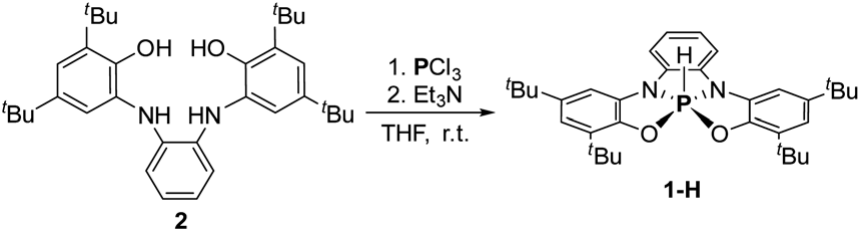
Synthesis of 1-H.

To prepare the geometrically constrained phosphoranide we decided to prepare phosphorane 1-H and to deprotonate it on the second step.^12^ Hydrophosphorane 1-H was prepared by the reaction of 2 with PCl_3_ and Et_3_N as base, in THF at room temperature (Scheme 1).

**Fig. 2:**
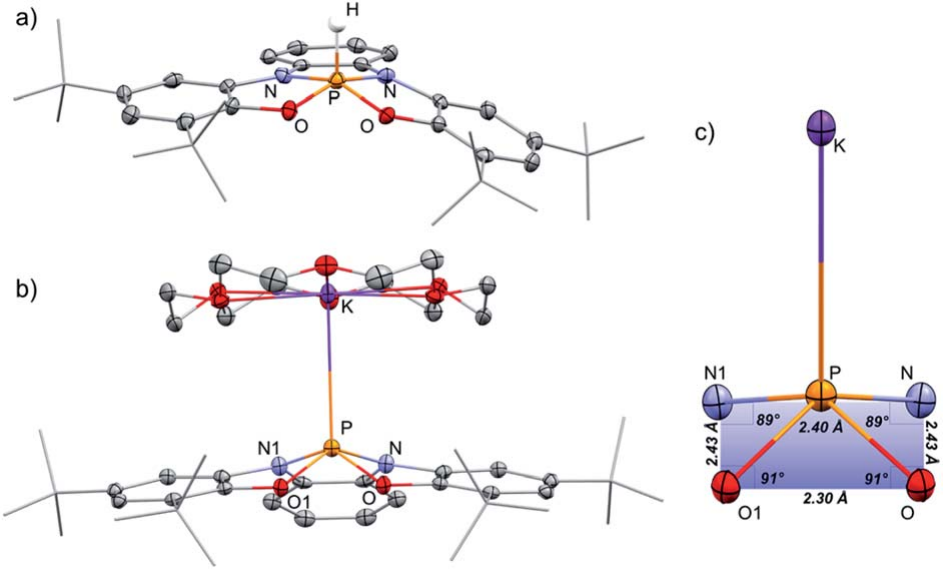
POV-ray depiction of 1-H (a), [1^−^][K(18-crown-6] (b), and the structural features of the SP fragment (c). Thermal ellipsoids at the 50% probability level, non-relevant hydrogens were omitted for clarity.

The ^31^P NMR spectrum of 1-H shows a doublet signal at *δ* = −40.21 ppm with one bond coupling *J*(PH) = 782 Hz. A corresponding signal in ^1^H NMR was found at *δ* = 8.79 ppm (*J*(HP) = 784 Hz). 1-H was isolated by crystallization from MeCN/CH_2_Cl_2_ mixture and its molecular structure was determined by X-ray crystallography (Fig. 2a). Most phosphoranes adopt a trigonal bipyramidal (TBP) geometry and undergo an intramolecular isomerization by a process known as Berry pseudorotation,^13^ which involves the interchange of the two axial ligands with two of the equatorial ones *via* an SP transition state. In contrast, 1-H adopts a distorted square pyramidal (SP) geometry, where two nitrogen and two oxygen atoms span the base of the pyramid, and the hydrogen occupies the apical position. The SP geometry in the case of 1-H is stabilized due to the rigidity of the tetra-anionic ONNO ligand (2).^14^ A small number of phosphoranes with an SP geometry is known in literature.^15*a–f*^

**Scheme 2:**
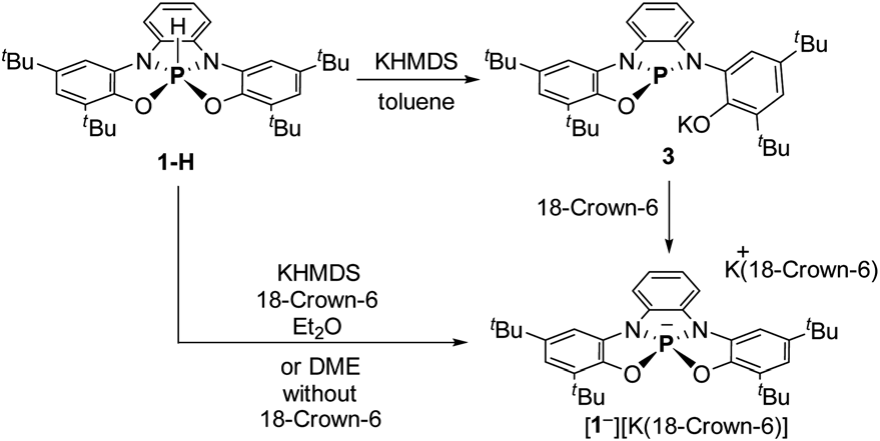
Synthesis of phosphoranide 1^−^.

To obtain 1^−^, 1-H was deprotonated with one equiv. of KHMDS in toluene. The ^31^P NMR of the reaction mixture showed a new signal at *δ* = 120.44 ppm, which clearly points to the formation of tricoordinated P^III^ species, meaning that one of the phenolic arms of the ligand dissociated from P center, giving 3 (Scheme 2). To transform 3 to the desired 1^−^, 18-crown-6 was added to the reaction mixture leading to the shift of the ^31^P NMR signal upfield to 73.96 ppm, which was attributed to the ^31^P chemical shift of 1^−^. After the optimization of this reaction, we found that the best conditions to prepare 1^−^ (60.11%) is the deprotonation of 1-H with KHMDS in presence of 18-crown-6 in Et_2_O (Scheme 2). It is important to note that 1^−^ could be also obtained *in situ* (99% conversion) by deprotonation of 1-H with KHMDS in dimethoxyethane (DME) without the addition of 18-crown-6 (see ESI† for more details).

[1^−^][K(18-crown-6)] was isolated by crystallization from Et_2_O by slow evaporation and its molecular structure was determined by X-ray crystallography (Fig. 2b). The P–K distance in [1^−^][K(18-crown-6] is 3.59 Å, which is longer than a typical P–K bond length (3.0–3.4 Å),^16*a,b*^ and similar to the P⋯K distance in a previously reported P/K(18-crown-6) salt.^16*c*^ The sum of angles around P center in 1^−^ is 332°, considerably lower than in 1-H (350°) due to the repulsion caused by the lone pair of electrons located at the phosphorus center. Remarkably, phosphoranide 1^−^ is of an SP geometry, which is an unprecedented geometry for phosphoranides, which to the best of our knowledge all have TBP geometry.^10*a–j*^ The SP geometry of 1^−^ is supported by the analysis of the X-ray molecular structure (Fig. 2c). Thus, the base of the pyramid in 1^−^, is only slightly distorted from a square geometry (∼4% distortion magnitude), this is due to different lengths between the O⋯O and N⋯N sides of the OONN square (Fig. 2c), and the height of the pyramid (from P to the base) in 1^−^ is 0.63 Å.

The SP geometry of 1^−^ is also confirmed by the analysis developed by Addison and co-workers.^17^ This analysis is used to ascertain the distortion magnitude between TBP and SP geometries, in this method the parameter *τ* is defined as (β-α)/60, where α and β are the longest and second longest angles respectively.^18^ This way *τ* can assume any number between 0 and 1, 0 being an ideal SP geometry and 1 an ideal TBP geometry. The *τ* of 1^−^ was calculated based on the angles α (O1–P–N = 139°) and β (O–P–N1 = 139°) and equals to 0, meaning an SP geometry of 1^−^. Based on all these data it can be concluded that 1^−^ is only slightly distorted from the perfect SP geometry. Noteworthy, previously reported example of geometrically constrained phosphoranide with the highest magnitude of distortion form TBP^10*h*^ is still only slightly distorted from the typical TBP geometry with *τ* = 0.85 calculated by Addison's method. It is also important to emphasize once again that despite the fact that the distortion from TBP to SP is a continuum, the SP on this continuum in most cases is a transition state of the Berry pseudorotation.^13^ This explains the very limited number of isolated SP phosphoranes^15*a–f*^ as well as previously unknown SP phosphoranides in which the lone pair of electrons destabilizes further the SP geometry due to increased repulsion.

**Scheme 3:**
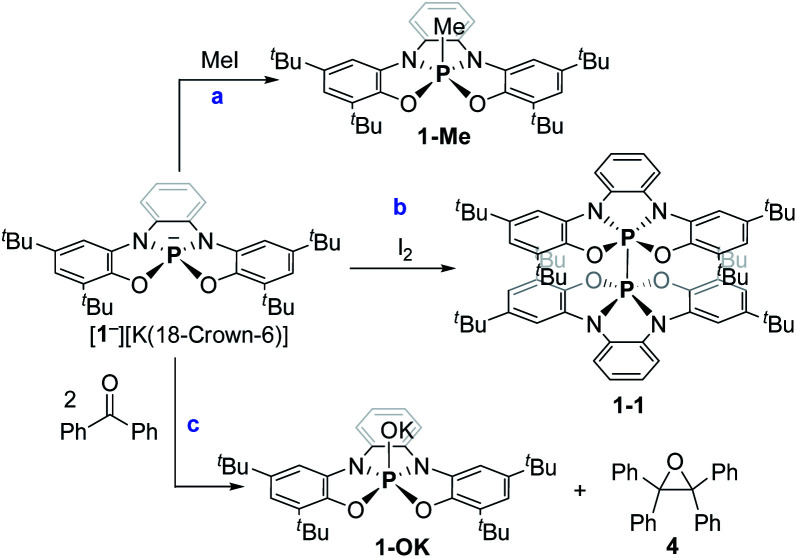
Preliminary reactivity study of phosphoranide 1^−^.

**Fig. 3:**
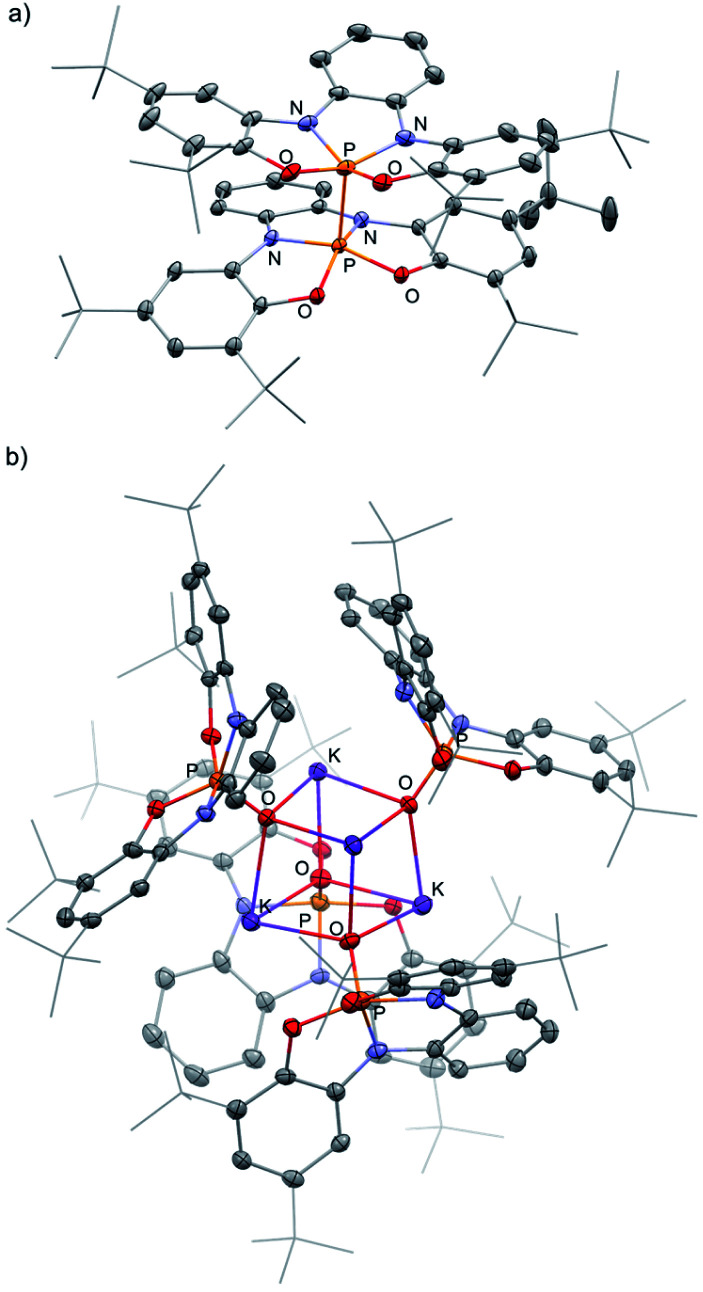
POV-ray depiction of 1-1 (a) and [1-OK]_4_ (b). Thermal ellipsoids at the 50% probability level, hydrogens were omitted for clarity.

Preliminary reactivity studies of 1^−^ were carried out. First, 1^−^ was reacted with MeI giving 1-Me (Scheme 3a; see ESI† for X-ray molecular structure of 1-Me). The reaction of 1^−^ with I_2_, however, produced, instead of the expected 1-I, dimer 1-1 (Scheme 3b), which was isolated by crystallization and its molecular structure was determined using X-ray crystallography (Fig. 3a). This reaction contrasts the reactivity of previously reported TBP phosphoranides that react in halogenation reactions producing halophosphoranes.^10*i,j*^ Importantly, 1-1 is a member of a P^V^–P^V^ dimeric type compounds that attracted much interest from the phosphorus community.^19*a–c*^ The P–P bond length in 1-1, of 2.23 Å, is in the range of P–P bonds in neutral P^V^–P^V^ type compounds that were previously reported.^19*a–c*^ As well as, P–P bond dissociation energy (BDE) in 1-1 was density functional theory (DFT) calculated^20*a*^ to be of Δ*H* = 68.27 and Δ*G* = 46.54 kcal mol^−1^, which is also in the range of BDEs of previously reported P^V^–P^V^ bonds.^19*a–c*^

Interestingly, 1^−^ reacted with 2 equiv. of benzophenone (Ph_2_CO) selectively leading to tetraphenyl epoxide (4) and phosphorane hydroxide 1-OK (Scheme 3c). 4, the tetramer of 1-OK ([1-OK]_4_) and its hydrolyzed product 1-OH, were crystallized and fully characterized (see Fig. 3b for X-ray molecular structure of [1-OK]_4_ and ESI for X-ray molecular structure of 4 and 1-OH). Noteworthy, typical TBP phosphoranides react with C=O bonds giving products of classical nucleophilic addition to carbonyls,^10*f*^ while this type of self-condensation reaction of carbonyls leading to oxiranes using phosphoranides is unprecedented. Similar type of reactivity, however, was reported for triaminophosphines.^21*a–d*^

While the reactivity of 1^−^ with MeI (Scheme 3a) is most probably of a nucleophilic nature, the reactivity of 1^−^ with I_2_ and formation of 1-1 points to the fact that 1^−^ reacts as a reductant in this reaction (Scheme 3b). The reactivity of 1^−^ with carbonyls (Scheme 3c) is more complicated and could either be of a nucleophilic type or a result of single electron transfer (SET) reaction.

**Fig. 4:**
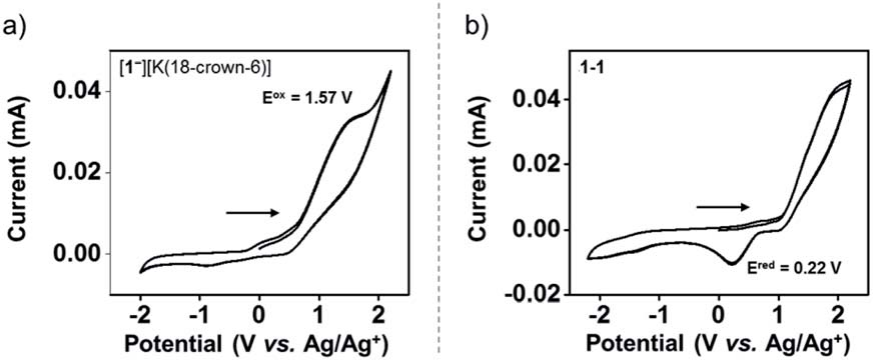
Cyclic voltammetry (CV) of [1^−^][K(18-crown-6)] (9.3 mM) (a) and 1-1 (3.4 mM) (b) in dry 0.1 M [*n*Bu_4_N][ClO_4_]/THF solution obtained at 0.1 V s^−1^ scan rate using glassy carbon electrodes, Pt wire, and Ag/Ag^+^ as the working, counter, and reference electrodes, respectively.

The redox chemistry of [1^−^][K(18-crown-6)] was studied by cyclic voltammetry (CV). The CV of [1^−^][K(18-crown-6)] (9.3 mM) was collected at a scan rate of 0.1 V s^−1^. [*n*Bu_4_N][ClO_4_] (0.1 M) in anhydrous THF was used as a supporting electrolyte. The CV of [1^−^][K(18-crown-6)] revealed an irreversible oxidation event centered at *E*^ox^_peak_ = 1.57 V *vs.* the Ag/Ag^+^ redox couple (Fig. 4a). The irreversibility of the CV of [1^−^][K(18-crown-6)] suggests a significant structural reorganization upon oxidation, possibly producing a highly reactive phosphoranyl radical 1˙. To study this redox process further, we performed a CV experiment on 1-1 (3.4 mM) under the same conditions, which revealed an irreversible reduction event centered at *E*^red^_peak_ = 0.22 V *vs.* the Ag/Ag^+^ redox couple (Fig. 4b). Importantly, this event was not observed in the CV of [1^−^][K(18-crown-6)] (Fig. 4a), thus suggesting that no dimerization of 1˙ occurs under these conditions.

Both reactions with I_2_ and carbonyls (Scheme 3b and c), in the case of a SET mechanism also assume the formation of phosphoranyl radical 1˙ as a possible intermediate. In general, phosphoranyl radicals (R_4_P˙) assume a TBP geometry, in which the unpaired electron is usually located as an equatorial (phantom) ligand or a tetrahedral geometry, in which the unpaired electron is located in the σ* orbital.^22^ Phosphoranyl radicals (R_4_P˙) are short-lived species that decay *via* α- or β-scission processes, or in presence of a good electron acceptor *via* SET to give phosphonium ions.^23*a–f*^ Noteworthy, aliphatic spirophosphoranyl radicals that assume rigid TBP structures, are persistent enough to be observed by EPR spectroscopy at high temperatures.^24^

**Scheme 4:**
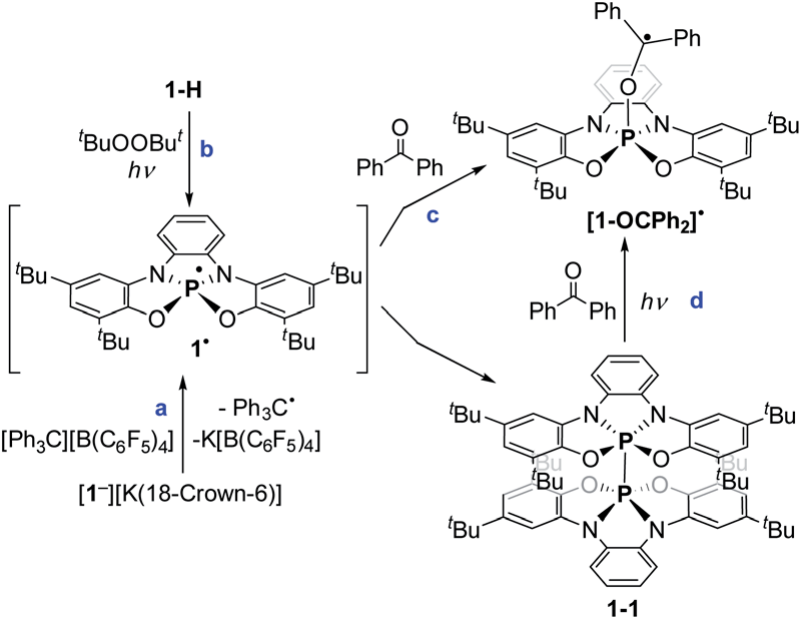
Generation of 1˙, its dimerization and trap with benzophenone.

**Fig. 5:**
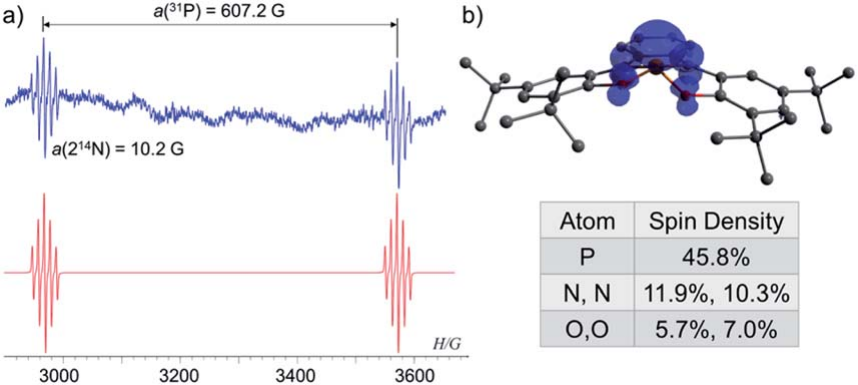
(a) EPR spectra of 1˙ (blue) and its simulation (red); (b) DFT calculated structure and Mulliken spin densities of 1˙.

To study the intermediacy of 1˙ and its chemistry further, we attempted the chemical oxidation of 1^−^ with [Ph_3_C][B(C_6_F_5_)_4_]. This reaction, however, immediately led to dimer 1-1 and Ph_3_C˙ (Scheme 4a), and 1˙ was not observed. This means that 1˙ is highly unstable, which could also be concluded from the irreversibility of the CV experiments (Fig. 4). However, unlike the electrochemical oxidation of 1^−^, its chemical oxidation leads to 1-1 through 1˙, which dimerizes faster than a sufficient concentration of it is formed to be measured by EPR spectroscopy. Importantly, unlike the typical dimerization decay pathway of phosphinyl radicals (R_2_P˙)^25*a,b*^ that could also be obtained from the oxidation reaction of the phosphinyl anions,^25*a,b*^ a dimerization decay pathway of phosphoranyl radicals had never been observed, thus the dimerization of 1˙ to 1-1 is an unprecedented decay pathway of phosphoranyl radicals. Due to inability to observe 1˙ in the oxidation reaction of 1^−^, we chose an alternative path to generate 1˙ and study it by EPR spectroscopy. Thus, 1-H was reacted with ^*t*^BuOOBu^*t*^ in benzene under UV irradiation (*λ* > 300 nm) at 330 K inside an EPRs' resonator cavity (Scheme 4b), and the EPR spectrum of 1˙ was recorded exhibiting a doublet of quintet [a(^31^P) = 607.2 G, a(2^14^N) = 10.2 G, *g* = 2.003] (Fig. 5). The signal disappeared immediately when the irradiation stopped, indicating a very short life-time of this radical. The structure of 1˙ was optimized using DFT,^20*b*^ and its EPR parameters were calculated.^20*c*^ The optimized structure of 1˙ assumes a slightly distorted SP geometry, which is unknown for phosphoranyl type radicals, with the spin density mostly localized on the P-center (Fig. 5). The calculated EPR ([a(^31^P) = 596.7 G, a(2^14^N) = 10.3 G, *g* = 2.003]), agree with the experimental data and support the formation of 1˙. The high reactivity of 1˙ can be explained by the exposed P-centered radical in an SP geometry.

It is important to note that a geometrical distortion was used recently to stabilize phosphorus anion radicals (R_3_P˙^−^) in a non-trigonal geometry.^26^ In contrast, in our case the geometrical distortion of the P-centered radical 1˙ causes the destabilization of the radical, and provides a new non-typical phosphoranyl radical decay route by dimerization.

**Fig. 6:**
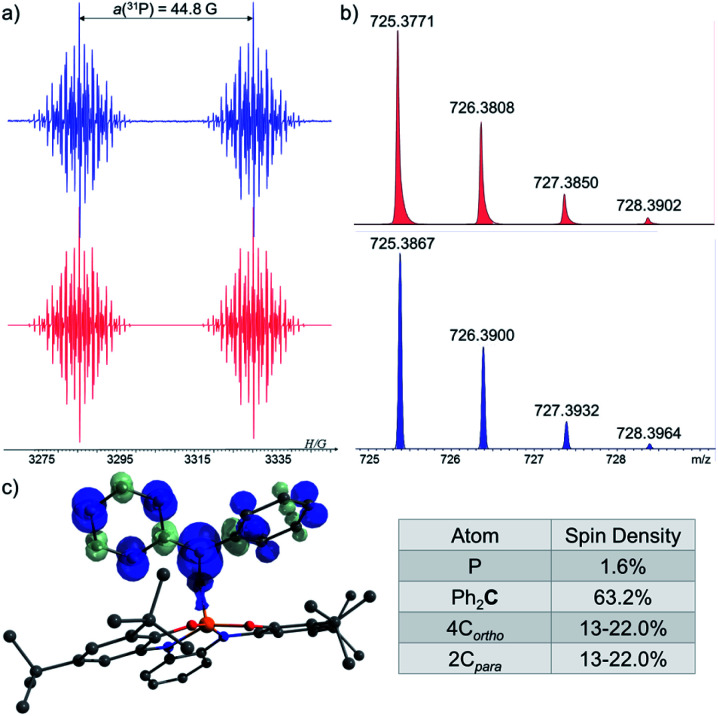
(a) EPR spectra of [1-OCPh_2_]˙ under UV-irradiation (*λ* > 300 nm) at 370 K (blue), and its simulation (red); (b) MS of the reduced [1-OCPh_2_]˙ (725.3771 (M)^−^) (red) and its simulation (blue); (c) DFT calculated Mulliken atomic spin densities in [1-OCPh_2_]˙.

In order to trap 1˙, a ^*t*^Bu-C_6_H_5_ solution containing 1-H, excess of ^*t*^BuOOBu^*t*^ and Ph_2_CO as a radical trap^27,28^ was irradiated with UV light (*λ* > 300 nm) for 20 min. at room temperature (see ESI†). As a result, a radical 1˙ adduct with benzophenone [1-OCPh_2_]˙ was formed (Scheme 4c) and measured by EPR spectroscopy, and its reduced form was detected by atmospheric pressure chemical ionization (APCI) MS in negative mode (725.3771 (M)^−^) (Fig. 6b). An EPR spectrum exhibited a doublet of multiplet [a(^31^P) = 44.8 G, a(4^1^H) =3.3 G, a(4^1^H) = 1.3 G, a(2^1^H) = 3.6 G, *g* = 2.003] (Fig. 6a). DFT calculations of the structure, spin density and EPR parameters^20*b*,*c*^ of [1-OCPh_2_]˙ [a(^31^P) = 43.1 G, a(4^1^H) = 4.4 G, a(4^1^H) = 2.3 G, a(2^1^H) = 4.5 G, *g* = 2.003], agree with the experimental data. Interestingly, when the irradiation was stopped, the EPR signal intensity dropped by ∼50%, indicating the decrease in radical concentration.

This behaviour is to be expected, since it is known that benzophenone-based C-centered radicals tend to dimerize through formation of weak C–C bonds. These dimers exist in equilibrium with the radicals that form them, and the stationary concentration of these radicals increases at elevated temperatures (above 300 K).^29*a,b*^ Indeed, heating the reaction mixture to 370 K led to the dissociation of the dimer and increase in intensity of the EPR signal corresponding to [1-OCPh_2_]˙. Importantly, the same radical ([1-OCPh_2_]˙) was generated when 1-1 was irradiated (*λ* > 300 nm) at 300 K in presence of Ph_2_CO, meaning that under these conditions 1-1 dissociates to 1˙,^30^ which is then trapped by Ph_2_CO (Scheme 4d).

**Scheme 5:**
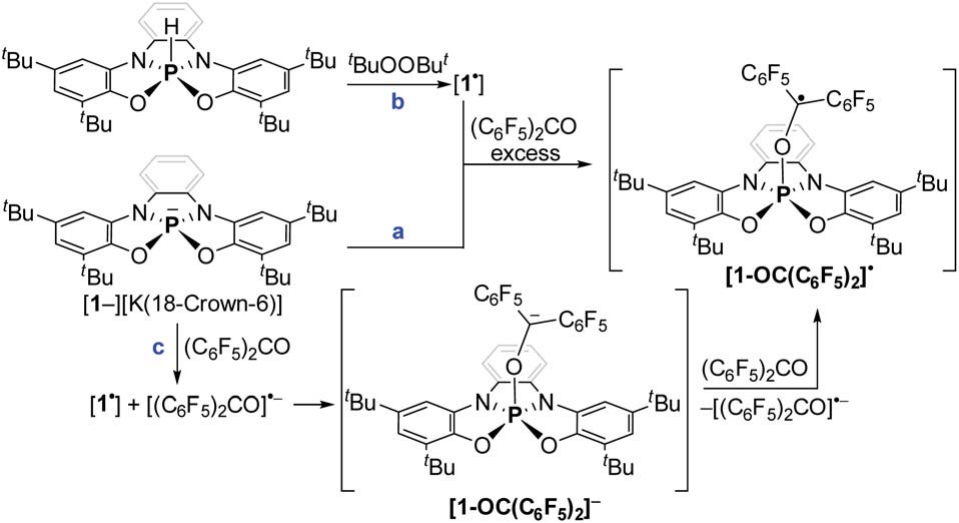
Generation of [1-OC(C_6_F_5_)_2_]˙ by two independent routes.

**Fig. 7:**
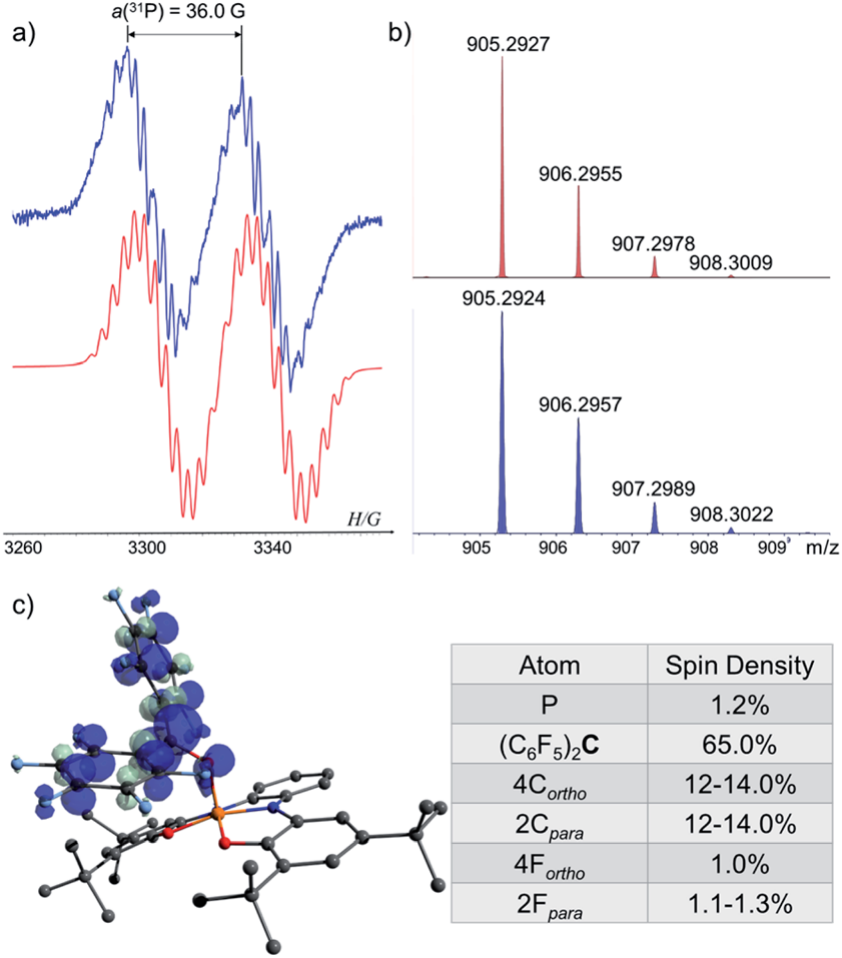
(a) EPR spectra of [1-OC(C_6_F_5_)_2_]˙ (blue) and its simulation (red); (b) MS of the reduced [1-OC(C_6_F_5_)_2_]˙ (905.2927 (M)^−^) (red) and its simulation (blue); (c) DFT calculated Mulliken atomic spin densities in [1-OC(C_6_F_5_)_2_]˙.

To study the reaction mechanism of 1^−^ with carbonyls (Scheme 4c) and the possibility of SET involvement in this mechanism, we performed the reaction of 1^−^ with excess of (C_6_F_5_)_2_CO, a stronger electron accepting analogue of Ph_2_CO, at r.t. (Scheme 5a) and studied this reaction by EPR spectroscopy. As a result, a doublet of multiplets (*g* = 2.004) having an hyperfine coupling constant (hfcc) with ^31^P nucleus a(^31^P) = 36.0 G and 10 non-equivalent ^19^F nuclei a(4^19^F-*o*) = 5.8, 3.7, 4.6, 4.1 G, a(4^19^F-*m*) = 3.6, 3.6, 2.8, 2.7 G and a(2^19^F-*p*) = 7.5, 6.1 G was recorded by EPR (Fig. 7a), which was attributed to radical [1-OC(C_6_F_5_)_2_]˙. Using APCI MS in negative mode, we were able to detect a mass that corresponds to the reduced radical [1-OC(C_6_F_5_)_2_]˙ (905.2927 (M)^−^) (Fig. 7b). The structure of [1-OC(C_6_F_5_)_2_]˙ was optimized, its spin density (Fig. 7c) and EPR parameters were calculated using DFT.^20*b*,*c*^ Expectedly, most of the spin density in [1-OC(C_6_F_5_)_2_]˙ resides on the central carbon [1-OC(C_6_F_5_)_2_]˙ (65%) with a significant delocalization into the strongly electron withdrawing C_6_F_5_ rings. The electron density on P is only around 1%, and the same goes to *ortho* and *para* fluorides (∼1%). The calculated *g*-value and *hfccs* are in good agreement with the experimental values (see ESI† for more details).

[1-OC(C_6_F_5_)_2_]˙ is possibly formed as a result of a SET reaction from 1^−^ to (C_6_F_5_)_2_CO giving transient 1˙ and [(C_6_F_5_)_2_CO]˙^−^,^31^ which couple to form anion [1-OC(C_6_F_5_)_2_]^−^ with the new P–O bond that is further oxidized by another equiv. of (C_6_F_5_)_2_CO (Scheme 5c). Importantly, [1-OC(C_6_F_5_)_2_]˙ is the trap product of 1˙ directly obtained from 1^−^ without irradiation, supporting the intermediacy of 1˙ in one electron oxidation reactions. Noteworthy, [(C_6_F_5_)_2_CO]˙^−^ is unstable and decomposes rapidly to various radical species,^31^ which complicated the outcome of the reaction between 1^−^ and (C_6_F_5_)_2_CO, leading to a complex mixture of products, however, the existence of the corresponding oxirane could still be detected in this mixture.

## Conclusions

In conclusion, we showed here the synthesis, isolation and structural characterization of the geometrically constrained SP phosphoranide 1^−^. A preliminary reactivity study showed that 1^−^ reacts with MeI, most probably as a nucleophile leading to 1-Me. In contrast, the reaction of 1^−^ with benzophenone involves a SET mechanism to give 1-O^−^ and the corresponding oxirane (4). In addition, 1^−^ reacts with I_2_ and Ph_3_C^+^*via* a SET mechanism producing unstable phosphoranyl radical 1˙, which immediately dimerizes to 1-1. 1˙ was independently generated by reaction with ^*t*^BuOOBu^*t*^ and its EPR spectrum was recorded. The structure of 1˙ was studied by DFT calculations and assumes an SP geometry, which is non-typical for phosphoranyl radicals, with the spin density mainly located at the P-center. 1˙ was trapped with benzophenone and (C_6_F_5_)_2_CO, and these radical adducts were studied by EPR, MS and DFT computations. We continue to study the chemistry of these fascinating geometrically constrained phosphorus-based ions.

## Data availability

All experimental procedures, spectral data, and computational data are available in the ESI.†

## Author contributions

S. V., I. M., D. B. and M. D. performed the synthetic work. D. B. analysed and solved all X-ray molecular structures. B. T. performed all EPR experiments. N. M. performed CV experiments. R. D. did all computational studies and wrote the manuscript with input from all authors.

## Conflicts of interest

The authors declare no conflict of interest.

## Supplementary Material

SC-013-D2SC01060G-s001

SC-013-D2SC01060G-s002
