# Supplementary material for: Geometrically constrained square pyramidal phosphoranide
Source: Chem Sci. 2022 Apr 27;13(20):5957–63. doi: 10.1039/d2sc01060g (PMC9132080; doi:10.1039/d2sc01060g)
Supplement: SC-013-D2SC01060G-s001 [file SC-013-D2SC01060G-s001.pdf]

Electronic Supplementary Material (ESI) for Chemical Science.
This journal is © The Royal Society of Chemistry 2022

Geometrically Distorted Square Pyramidal Phosphoranide

Solomon Volodarsky,[†] Irina Malahov,[†] Deependra Bawari, Mohanad Diab, Naveen Malik, Boris Tumanskii, and Roman Dobrovetsky*

Table of Contents

1. General experimental considerations....S3
2. X-ray Crystallography....S3
3. Experimental procedures....S3
4. Electrochemistry....S20
5. EPR experiments....S21
6. DFT Computations....S25
7. References....S37

1. General experimental considerations: All preparations were carried out under an anhydrous N_2 atmosphere using standard Schlenk and glove box techniques. All glassware was oven dried and cooled under vacuum before use. Commercial reagents were purchased from Sigma Aldrich, Strem or Apollo Scientific and used without further purification unless indicated otherwise. **2** was prepared following the reported procedures.[1] NMR spectra were recorded at room temperature using a Bruker AvanceIII-400 MHz spectrometer. Data for ^{1}H NMR are reported as follows: chemical shift (*δ* ppm), integration, multiplicity (s = singlet, d = doublet, t = triplet, q = quartet, quin = quintet, m = multiplet, br = broad), coupling constant (Hz), assignment.

2. X-ray Crystallography:

Single crystal X-ray diffraction data were collected on a Bruker KAPPA APEX II diffractometer equipped with an APEX II CCD detector using a TRIUMPH monochromator with a MoK*α* X-ray source (*α* = 0.710 73 Å). The crystals were mounted on a cryoloop with Paratone oil, and all data were collected at 100(2) K. Unit cell determination and refinement and data collection were done using the Bruker APPEX-II suite,[2] data reduction and integration were performed using SAINT v8.34A (Bruker, 2013)[3] and absorption corrections and scaling were done using SADABS-2014/5 (Bruker,2014/5)[4]. All the crystal structures were solved through OLEX2[5] package using SHELXT[6] and the structures were refined using SHELXL[6]. All non-hydrogen atoms were refined anisotropically. All the figures were generated using Mercury 3.10.2.

3. Experimental Procedures

Synthesis of 1-H:

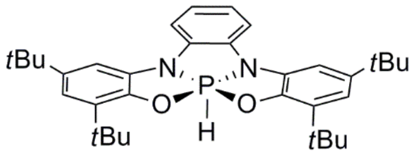

PCl_3 (0.47 mL, 5.34 mmol) was added to a THF (70 mL) solution of compound **2** (2.3 g, 4.45 mmol) at r.t., after 5 min followed by the addition of Et_3N (2.5 mL, 17.8 mmol). This mixture was stirred at r.t. overnight. The solution was filtered by cannula and thereafter all the volatiles were evaporated under vacuum. The light yellow solid was washed with acetonitrile (50 mL) that afforded white solid. This solid was crystallized by slow evaporation of acetonitrile-DCM mixture (3:1) at room temperature. Yield: 1.2 g (49.59 %).

^{1}H NMR (400 MHz, $CDCl_3$); *δ* = 8.79 (d, 1H, J_{H-P} = 784 Hz, -P*H*), 7.65 (m, 2H, Ar-*H*), 7.55 (s, 2H, Ar-*H*), 7.12 (m, 2H, Ar-*H*), 7.04 (s, 2H, Ar-*H*), 1.51 (s, 18H, -C(CH_3)$_3$), 1.43 (s, 18H, -C(CH_3)$_3$) ppm. **^{13}C NMR (100 MHz, $CDCl_3$); *δ*** = 144.12, 139.90, 133.60 (d, *J* = 9 Hz), 130.32 (d, *J* = 14 Hz), 130.05 (d, *J* = 20 Hz), 121.08, 116.19, 110.59 (d, *J* = 10 Hz), 107.20 (d, *J* = 9 Hz), 35.17 (-*C*(CH_3)$_3$), 34.71 (-*C*(CH_3)$_3$), 32.00 (-C(*C*H_3)$_3$), 29.72 (-C(*C*H_3)$_3$) ppm. **^{31}P NMR (162 MHz, $CDCl_3$); *δ*** = -40.21 (d, J_{P-H} = 782 Hz) ppm. **HRMS** (ES^+): Calculated for $C_{34}H_{46}N_2O_2P$: 545.3297 $[M+H]^+$; Obs: 545.3292. **EA**: Calc'd for $C_{34}H_{46}N_2O_2P$: C, 74.97; H, 8.33; N, 5.14; Obs: C, 74.91; H, 8.37; N, 5.12.

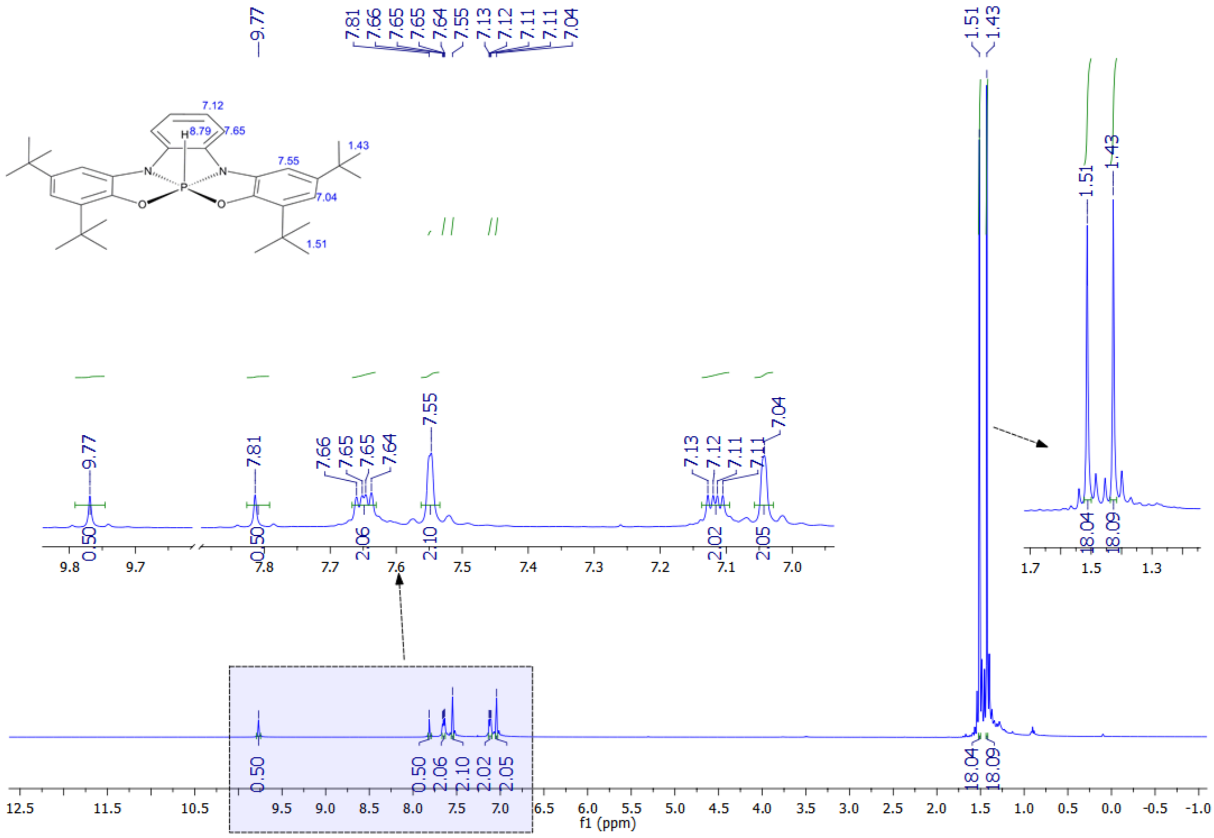

Figure S1. ^{1}H NMR spectrum (400 MHz, $CDCl_3$) of **1-H**.

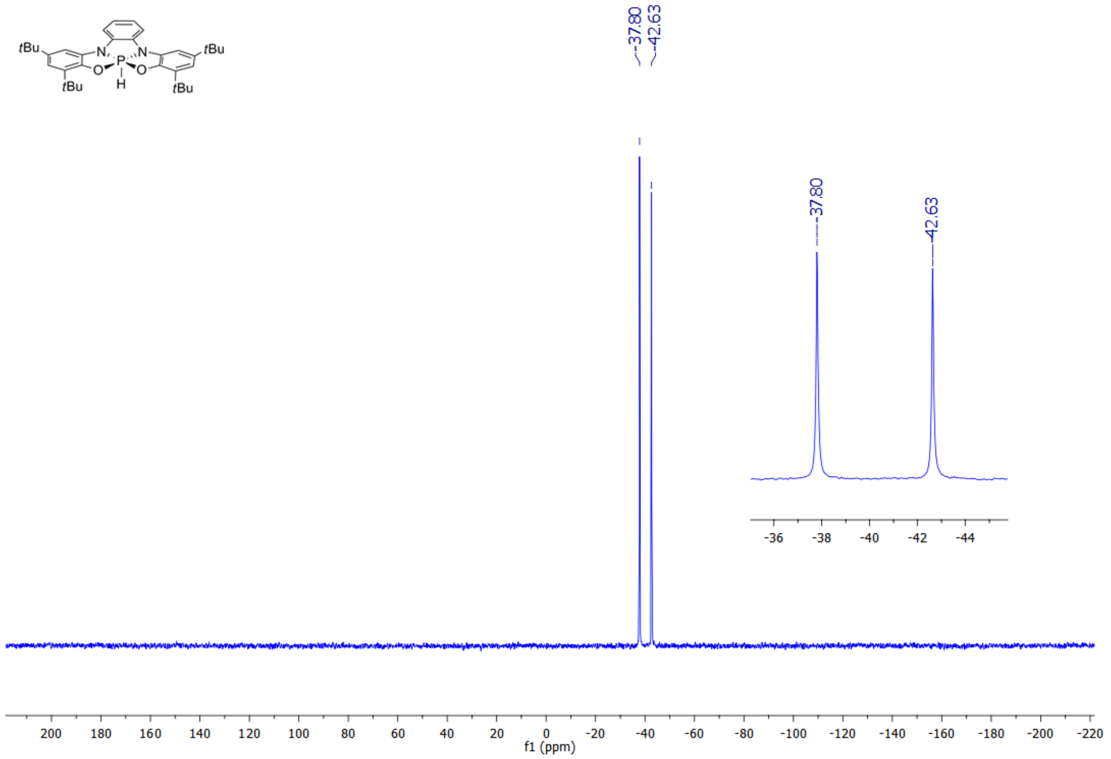

Figure S2. ^{31}P NMR spectrum (162 MHz, $CDCl_3$) of **1-H**.

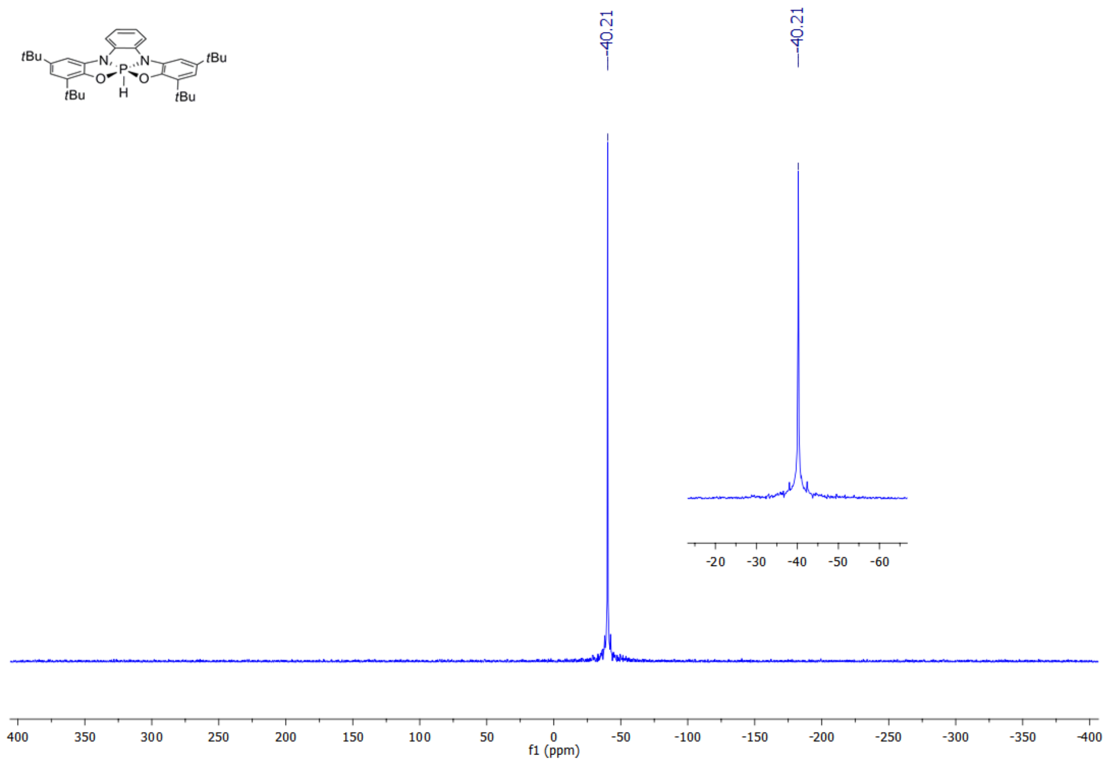

Figure S3. $^{31}P\{^{1}H\}$ NMR spectrum (162 MHz, $CDCl_3$) of **1-H**.

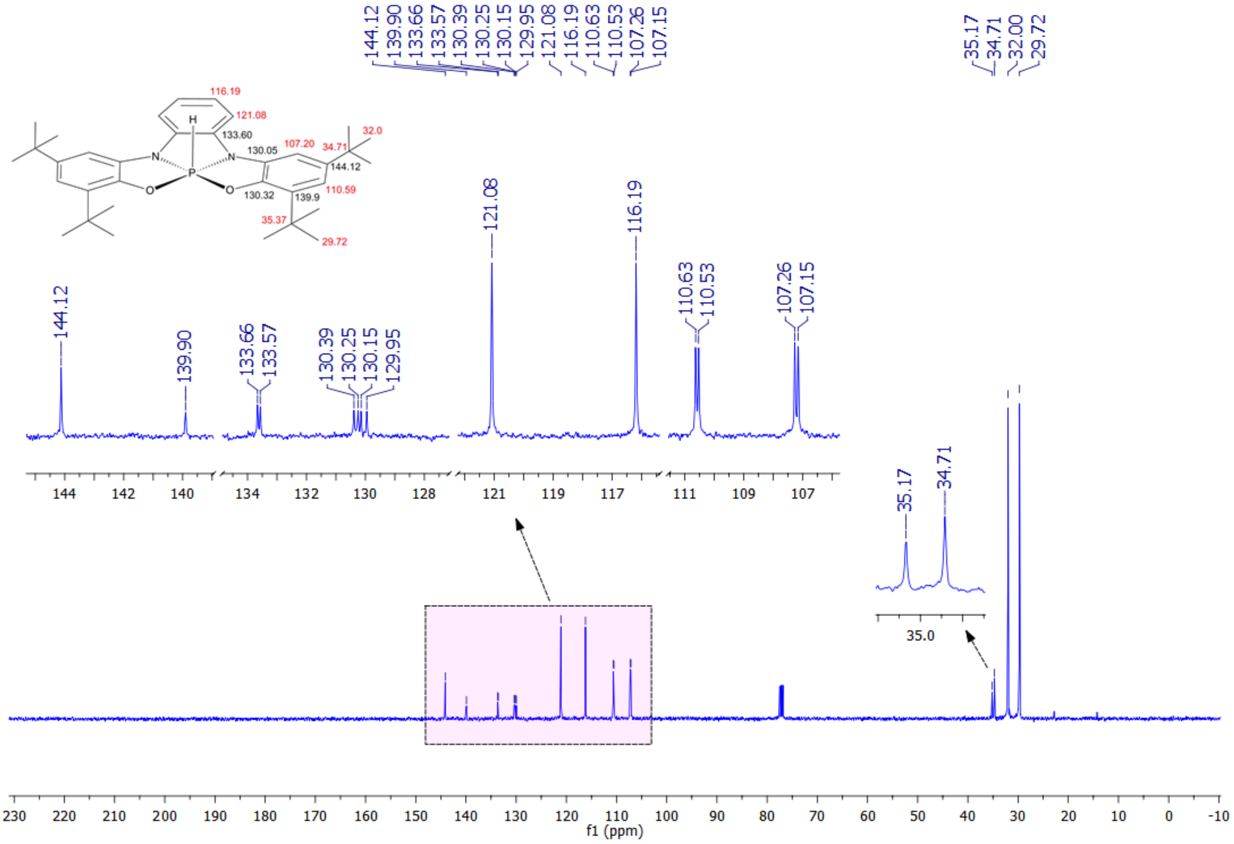

Figure S4. ^{13}C NMR spectrum (100 MHz, $CDCl_3$) of **1-H**.

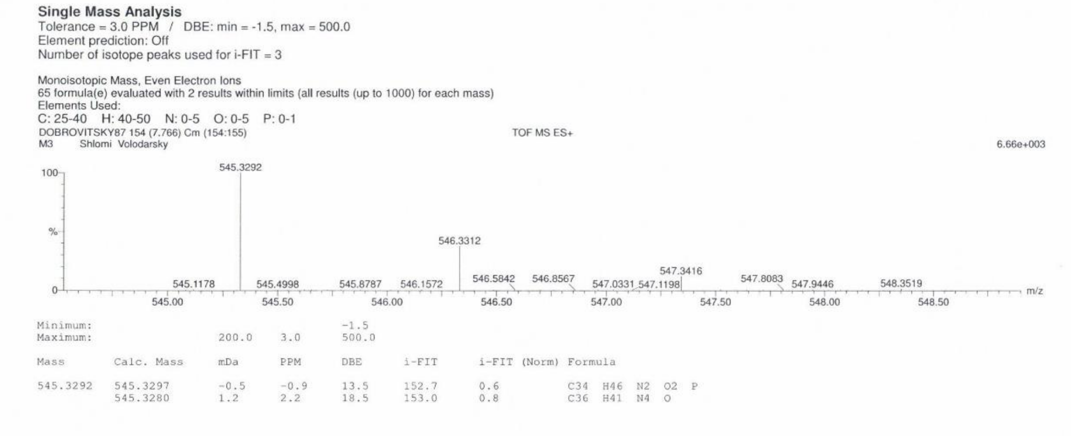

Figure S5. HRMS spectrum of **1-H**.

Synthesis of [1⁻][K(18-crown-6)]:

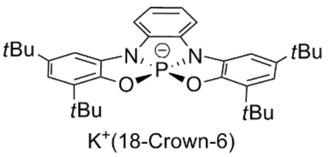

KHMDS (0.192 g, 0.964 mmol) was added to a 10 mL of Et_2O solution of compound **1-H** (0.5 g, 0.918 mmol) at room temperature. An instant reaction was observed (the reaction in toluene showed the formation of **3** by ^{31}P NMR spectrum (120.00 ppm).18-Crown-6 (0.243 g, 0.918 mmol) was added (***Note:*** complete evaporation of Et_2O showed decomposition). The slow evaporation of this solution at room temperature afforded colorless crystals of *[1⁻][K(18-crown-6)]* after one week. Yield: 0.3 g (38.6%). **^1H NMR (400 MHz, in d8-THF); *δ*** = 7.16 (m, 2H, Ar-*H*), 7.11 (s, 2H, Ar-*H*), 6.60 (s, 4H, Ar-*H*), 3.29 (s, 24H, -CH_2-crown ether),1.43 (s, 18H, -C(CH_3)$_3$), 1.31 (s, 18H, -C(CH_3)$_3$) ppm. **^{13}C NMR (100 MHz, in d8-THF); *δ*** = 147.89 (d, *J* = 10 Hz), 139.55 (d, *J* = 5 Hz), 138.70, 137.66 (d, *J* = 2 Hz), 131.20, 118.50, 112.94, 112.57, 107.13, 71.05 (-*C*H_2-crown ether), 35.29 (-*C*(CH_3)$_3$), 32.78 (-C(*C*H_3)$_3$), 30.48 (-C(*C*H_3)$_3$) ppm. **^{31}P NMR (162 MHz, in d8-THF); *δ*** = 74.40 ppm.

Insitu formation of 1⁻ in DME: KHMDS (0.192 g, 0.964 mmol) was added to 8 mL DME solution of compound **1-H** (0.5 g, 0.918 mmol) at room temperature. An instant reaction was observed that showed the formation of **1⁻** by multinuclear NMR.

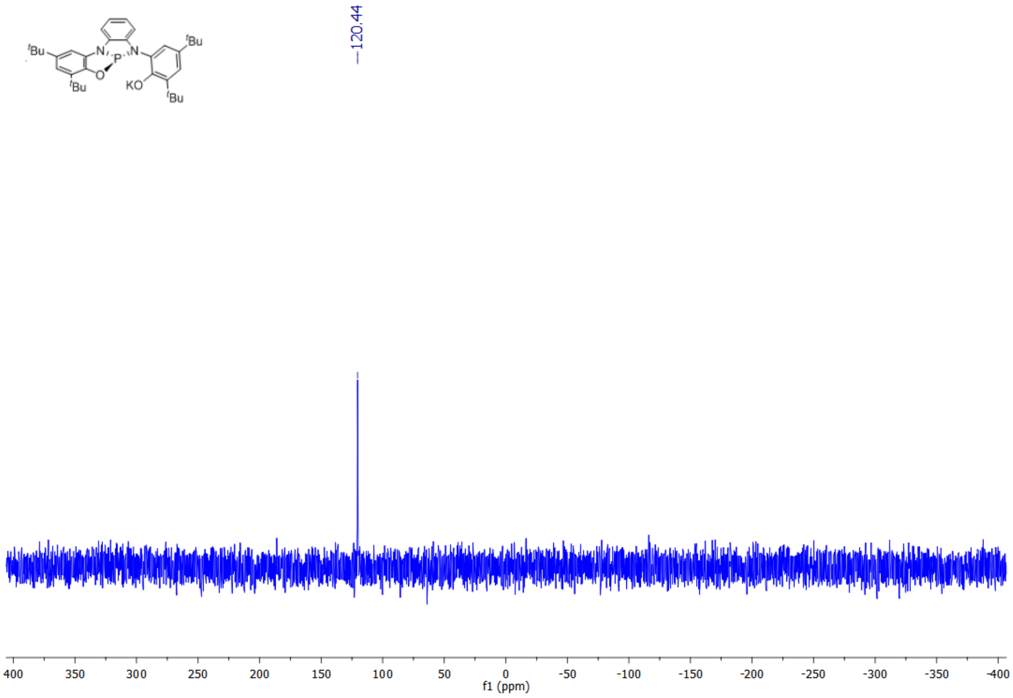

Figure S6. In-situ ^{31}P NMR spectrum (162 MHz, in toluene) of **3.**

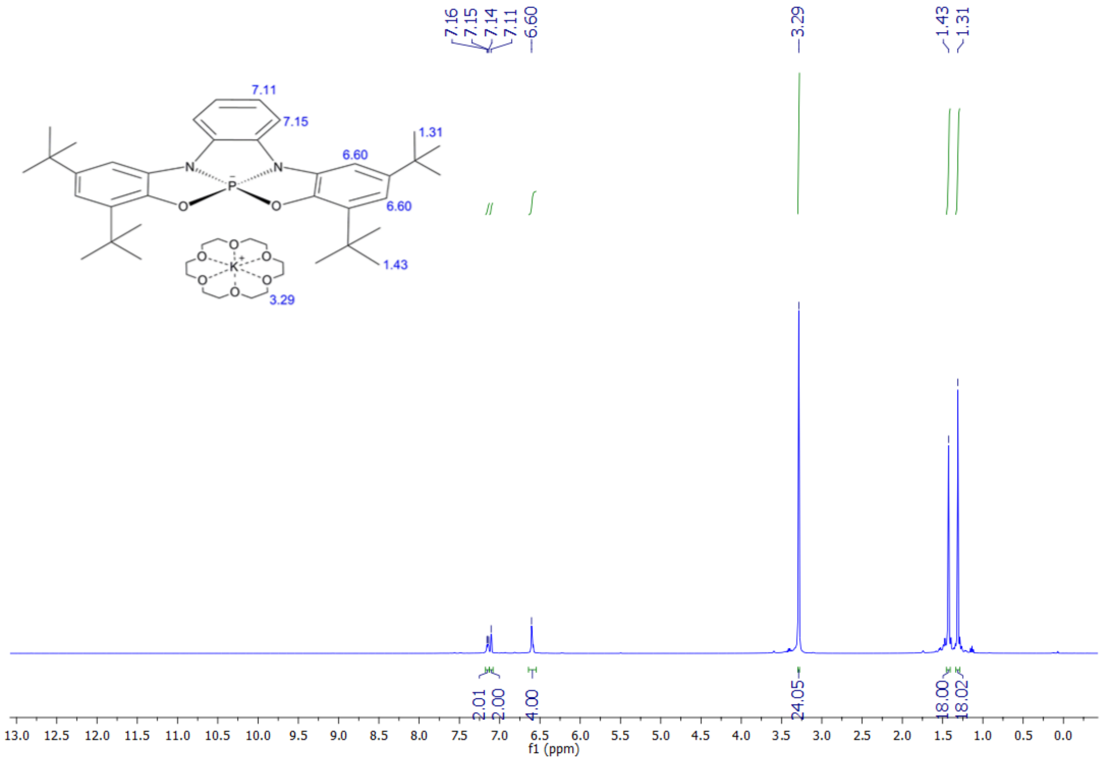

Figure S7. ^{1}H NMR spectrum (400 MHz, d8-THF) of **[1^{-}][K(18-crown-6)].**

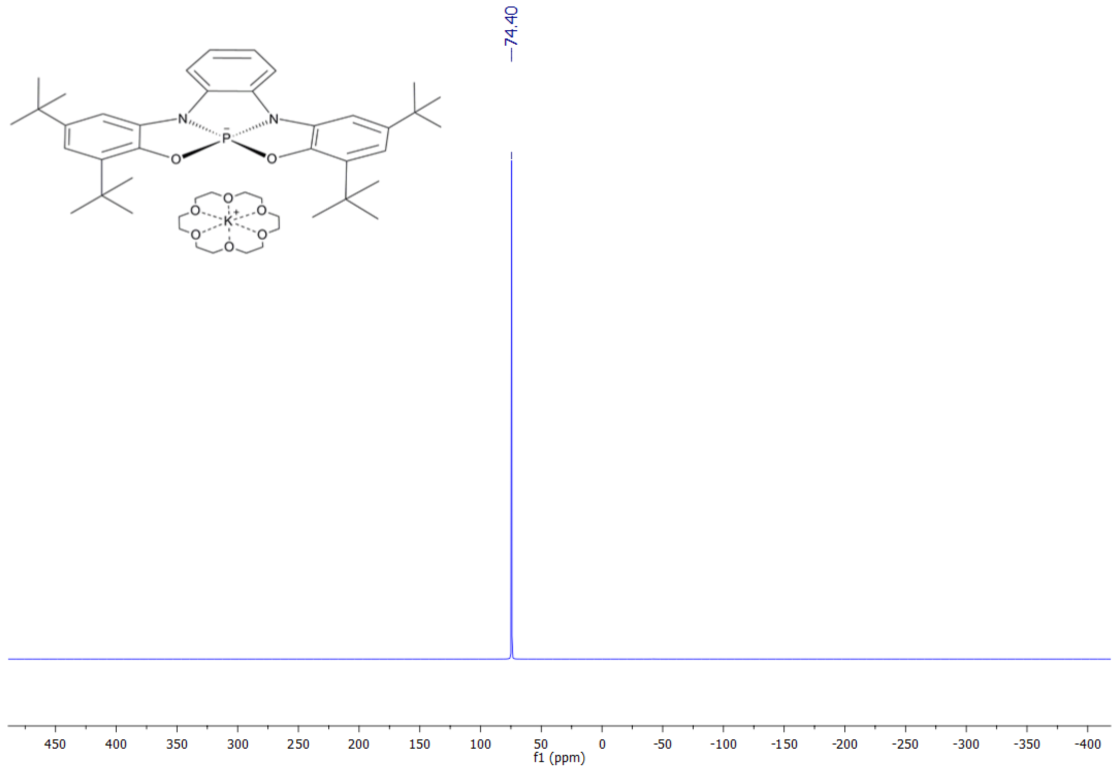

Figure S8. ^{31}P NMR spectrum (162 MHz, d8-THF) of **[1^{-}][K(18-crown-6)].**

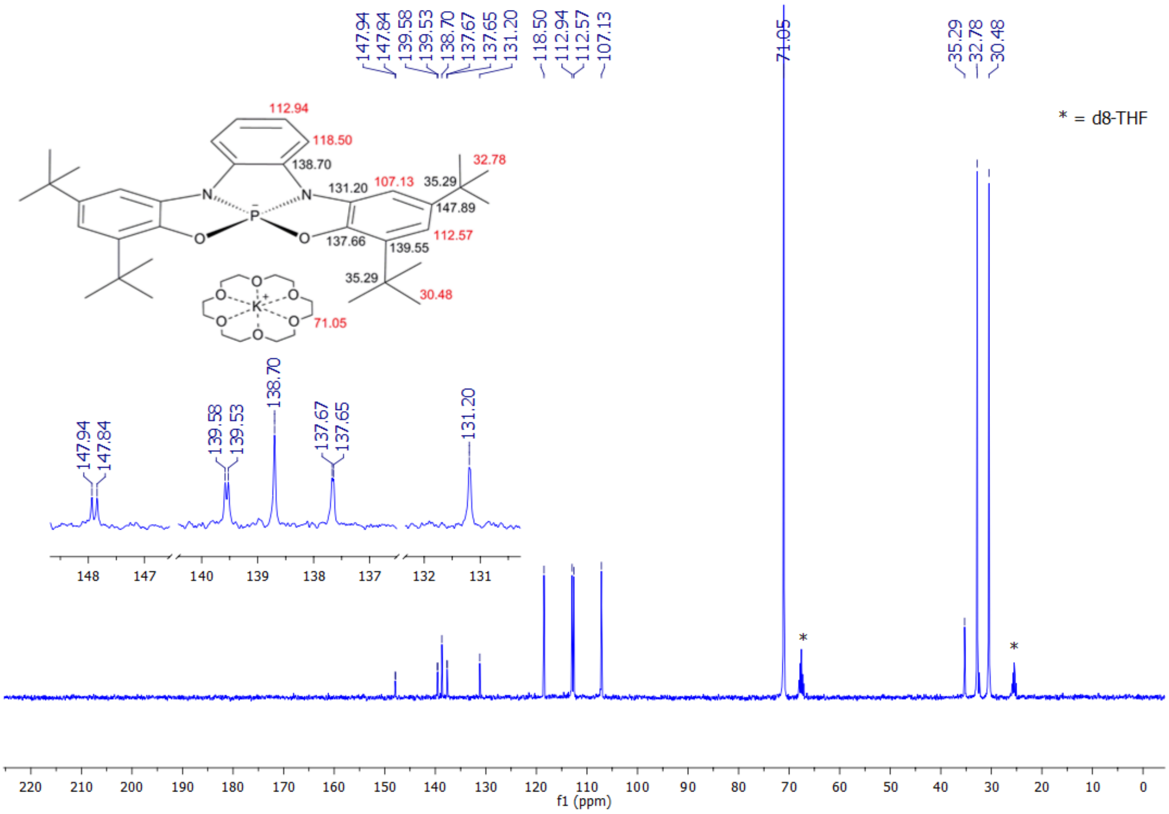

Figure S9. ^{13}C NMR spectrum (100 MHz, d8-THF) of **[1^{-}][K(18-crown-6)].**

Synthesis of 1-1:

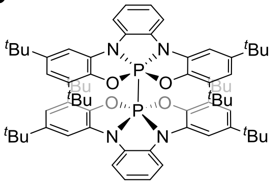

[1^{-}][K(18-crown-6)] (0.333 g, 0.394 mmol) was added to a 10 mL Et_2O solution of Iodine (100 mg, 0.394 mmol) at room temperature. An instant reaction was observed. The solution was filtered and all the volatiles were evaporated under vacuum (to remove iodine the compound was also heated to 80 °C). The obtained solid was dissolved in hexane (4 mL) and few drops of acetonitrile were added. Slow evaporation of this solution at room temperature afforded colorless crystals after two weeks. Yield: 0.12 g (56.0%).

Alternative path: Similar results were also obtained on performing the reaction in DME solution, through in situ generation of **1**$^{-}$ using KHMDS and without 18-crown-6. Yield: 0.13 g (65.12%). **^{1}H NMR (400 MHz, $CDCl_3$, at - 40 °C); *δ*** = 7.57 (s, 2H, Ar-*H*), 7.21 (s, 2H, Ar-*H*), 6.91 (s, 4H, Ar-*H*), 6.83 (s, 2H, Ar-*H*), 6.68 (s, 4H, Ar-*H*), 6.59 (s, 2H, Ar-*H*), 1.43 (s, 18H, -C(CH_3)$_3$), 1.33 (s, 18H, -C(CH_3)$_3$), 1.21 (s, 18H, -C(CH_3)$_3$), 0.87 (s, 18H, -C(CH_3)$_3$) ppm. **^{13}C NMR (100 MHz, $CDCl_3$, at - 40 °C); *δ*** = 142.87, 141.70, 139.75 (d, *J* = 13 Hz), 132.64, 132.22, 129.72 (d, *J* = 13 Hz), 128.80 (d, *J* = 18 Hz), 120.39, 119.59, 116.01, 115.08, 111.98, 110.73, 107.48, 106.34, 34.88 (-*C*(CH_3)$_3$), 34.78 (-*C*(CH_3)$_3$), 34.44 (-*C*(CH_3)$_3$), 34.00 (-*C*(CH_3)$_3$), 31.90 (-C(*C*H_3)$_3$), 31.81 (-C(*C*H_3)$_3$), 29.69 (-C(*C*H_3)$_3$), 28.91 (-C(*C*H_3)$_3$) ppm. **^{31}P NMR (162 MHz, $CDCl_3$, at - 40 °C); *δ*** = -31.70 ppm. **HRMS** (AP^{+}): Calculated for $C_{66}H_{89}N_4O_4P_2$: 1087.6359 [M+H]$^{+}$; Obs: 1087.6348. **EA**: Calc'd for $C_{68}H_{88}N_4O_4P_2$·($4H_2O$+$1.5O_2$): C, 67.64; H, 8.01; N, 4.64; Obs: C, 67.11; H, 7.99; N, 4.62. Only partially hydrolyzed and oxidized **1-1** could be obtained after multiple attempts.

Note: ^{1}H and ^{13}C NMR spectra of **1-1** at room temperature showed broad merged signals. We believe that the broaden NMR spectra is a result of London dispersion forces between the large substituents at the two P-centers, that preclude free rotation around P-P bond and the rotational vibration affects the NMR spectra. At low temperature we diminish the effect of this vibration and get a better resolved spectra, but it is important to mention that we still get four different t-Bu signals that supports this explanation. A spontaneous dissociation at RT is not observed by EPR.

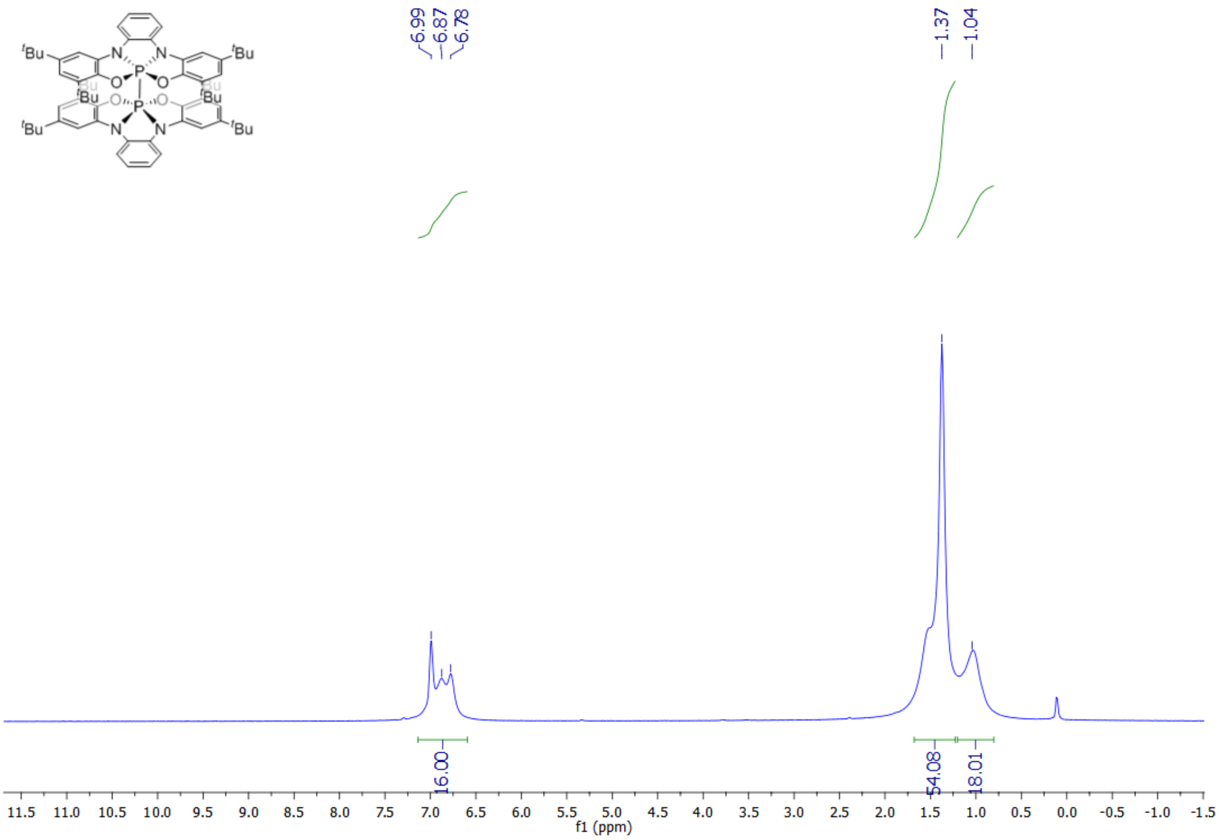

Figure S10. ^{1}H NMR spectrum (400 MHz, $CDCl_3$) of **1-1** at room temperature.

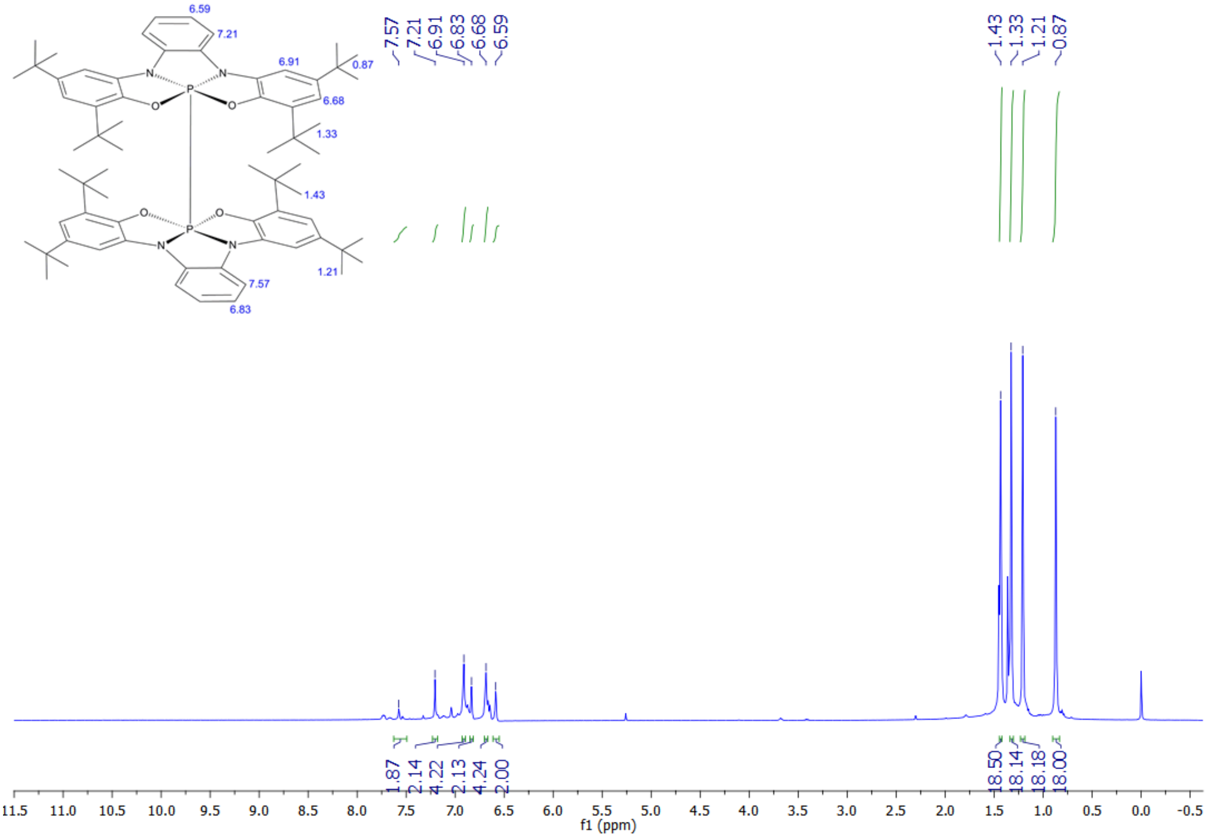

Figure S11. ^{1}H NMR spectrum (400 MHz, $CDCl_3$) of **1-1** at -40 °C**.**

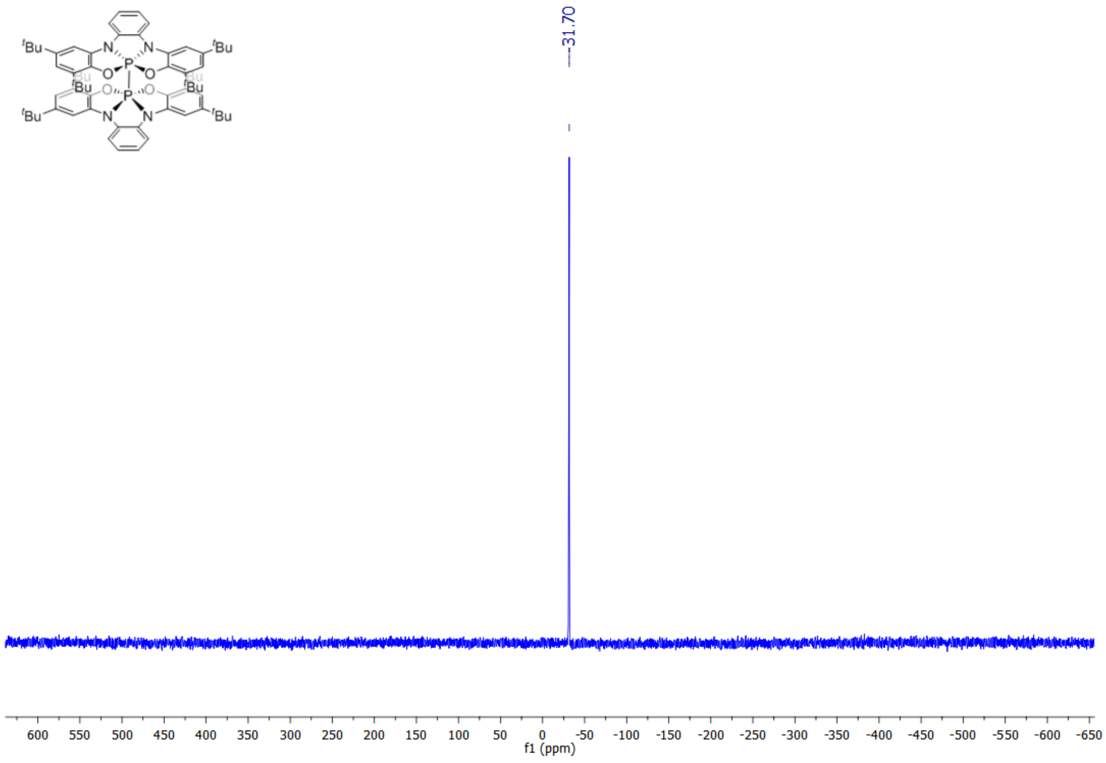

Figure S12. ^{31}P NMR spectrum (162 MHz, $CDCl_3$) of **1-1.**

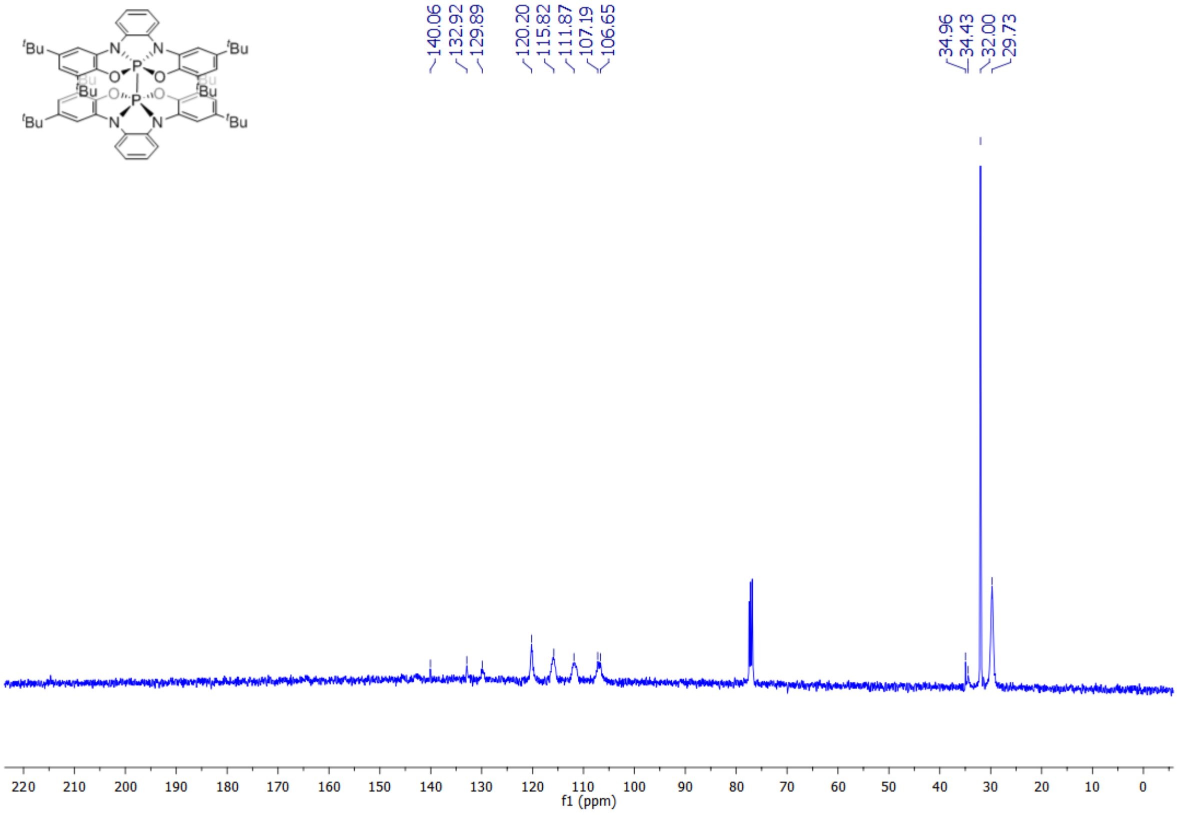

Figure S13. ^{13}C NMR spectrum (100 MHz, $CDCl_3$) of **1-1** at room temperature**.**

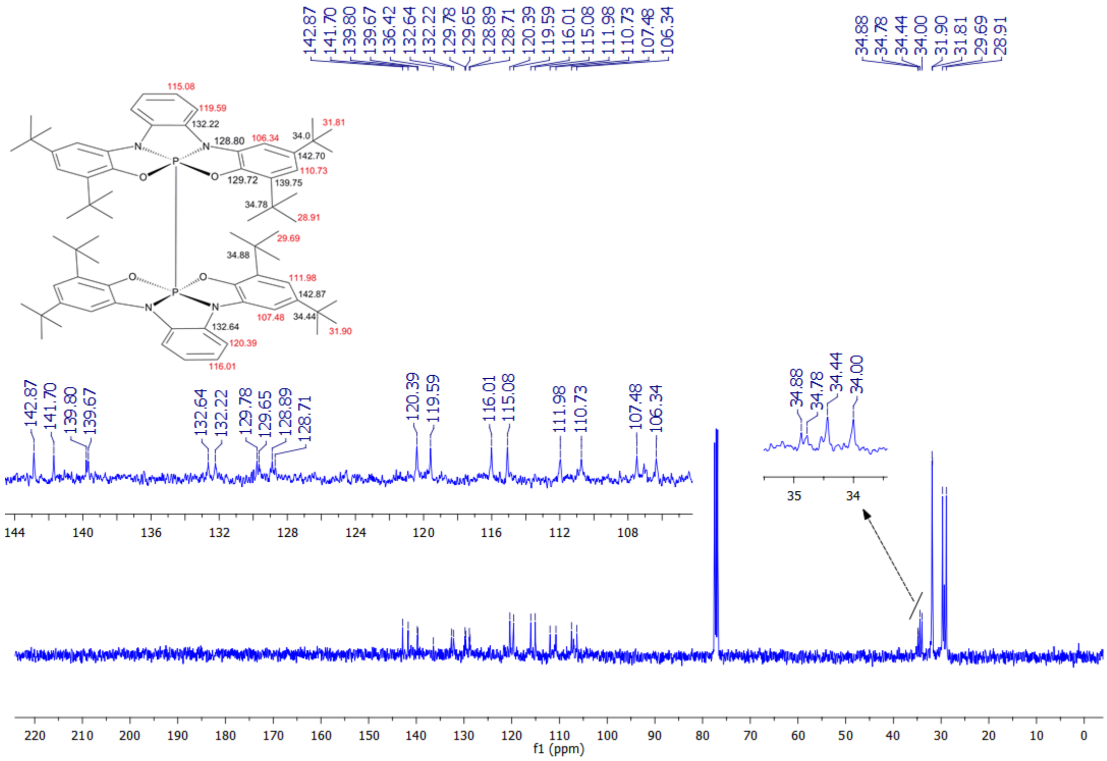

Figure S14. ^{13}C NMR spectrum (100 MHz, $CDCl_3$) of **1-1** at -40 °C**.**

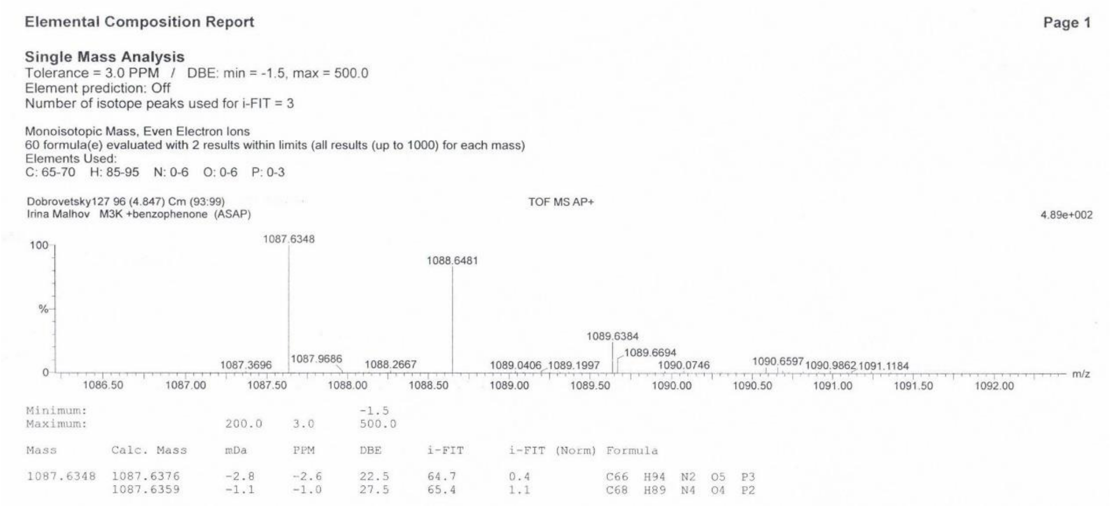

Figure S15. HRMS spectrum of **1-1.**

Synthesis of 4 and 1-O^{-}:

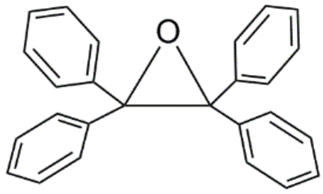

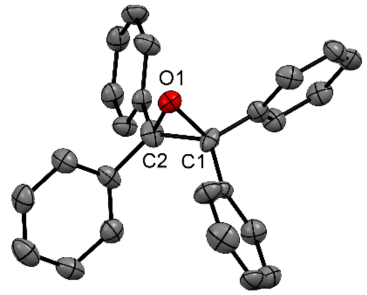

Molecular structure of **4**

[1^{-}][K(18-crown-6)] (0.265 g, 0.313 mmol) was added to a 8 mL Et_2O solution of benzophenone (114 mg, 0.626 mmol) at room temperature. An instant reaction was observed. All the volatiles were evaporated in vacuum. The obtained solid was dissolved in acetonitrile (4 mL) which left to crystalize at -30 °C, after 2 days affording colorless crystals of **4** (yield: 45 mg, 41.3%). The decanted solution was also evaporated and recrystallized in 5 mL hexane solution that on slow evaporation at room temperature after three days afforded colorless crystals of **1-OK** (Yield: 91 mg, 48.6 %).

Alternative path: The formation of **4** (yield: 51 mg, 46.9%) and **1-OK** (Yield: 115 mg, 61.5%) could also be observed on performing the reaction in a DME solution, that proceeds through in situ generation of **1**$^{-}$ using KHMDS and without 18-crown-6. (Note: Crystals of hydrolyzed product, **1-OH** were also obtained during repeated attempts to crystallized **1-OK**)

4: ^{1}H NMR (400 MHz, $CDCl_3$); *δ* = 7.23 (m, 8H, Ar-*H*), 7.11 (m, 12H, Ar-*H*) ppm. **^{13}C NMR (100 MHz, $CDCl_3$); *δ*** = 138.76, 128.37, 127.70, 127.20, 74.18 (*C*-O) ppm. **HRMS** (ES^{+}): Calculated for $C_{26}H_{20}O$: 348.1514 $[M+]^{+}$; Obs: 348.1516.

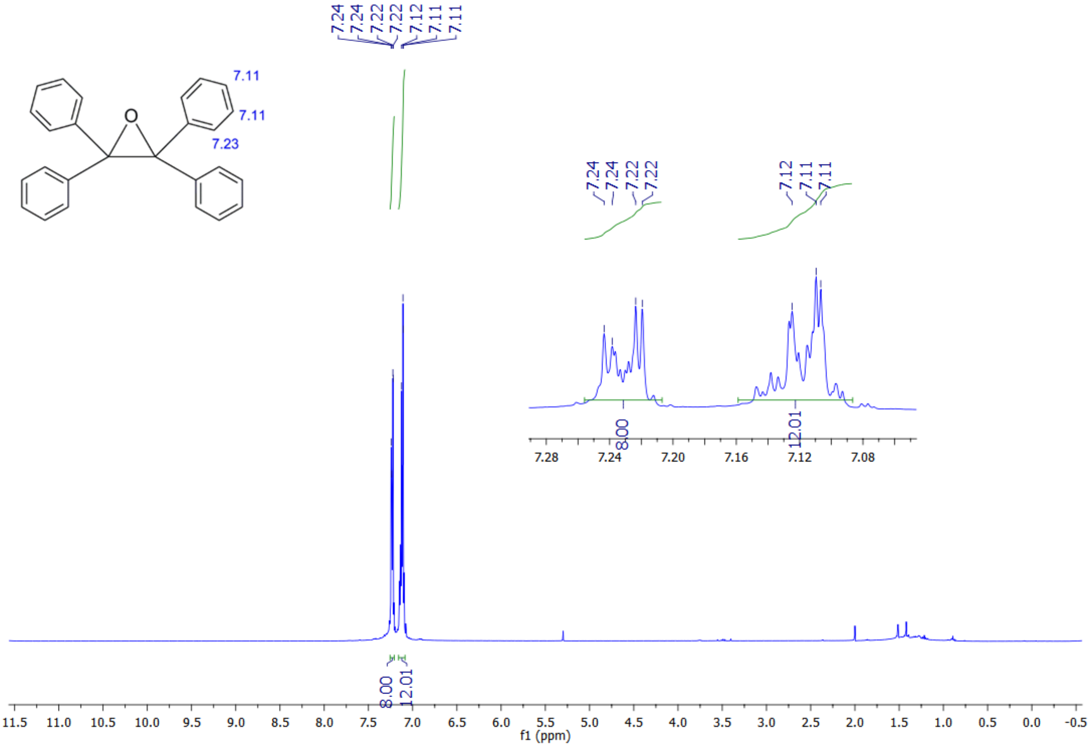

Figure S16. ^{1}H NMR spectrum (400 MHz, $CDCl_3$) of **4.**

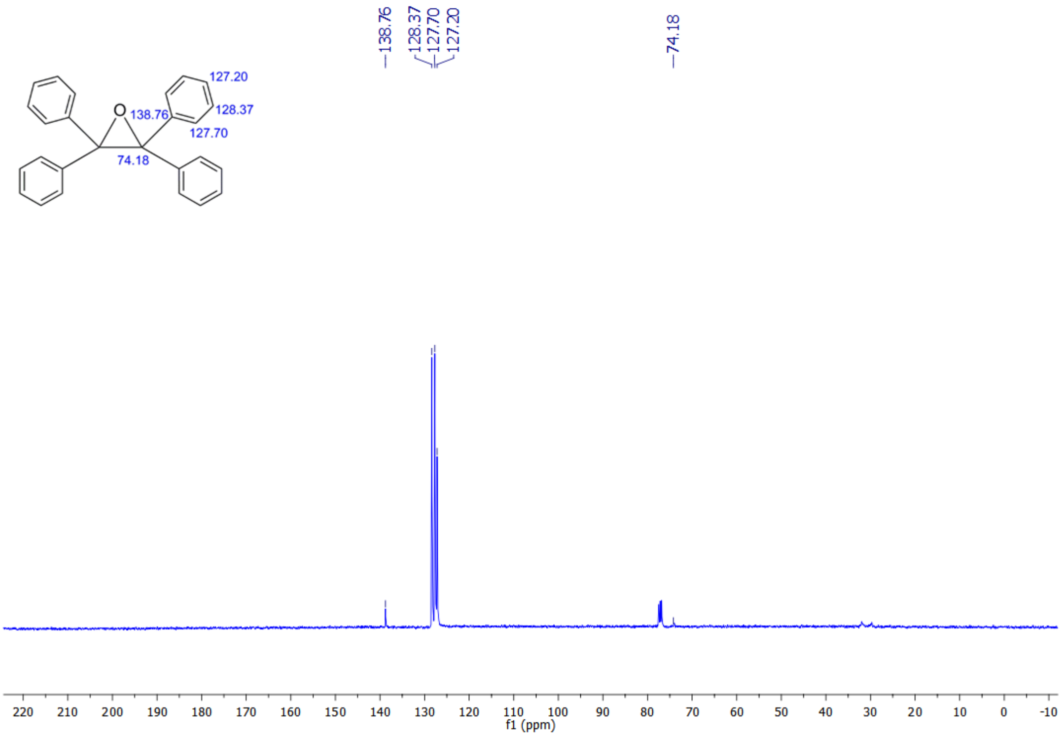

Figure S17. ^{13}C NMR spectrum (400 MHz, $CDCl_3$) of **4.**

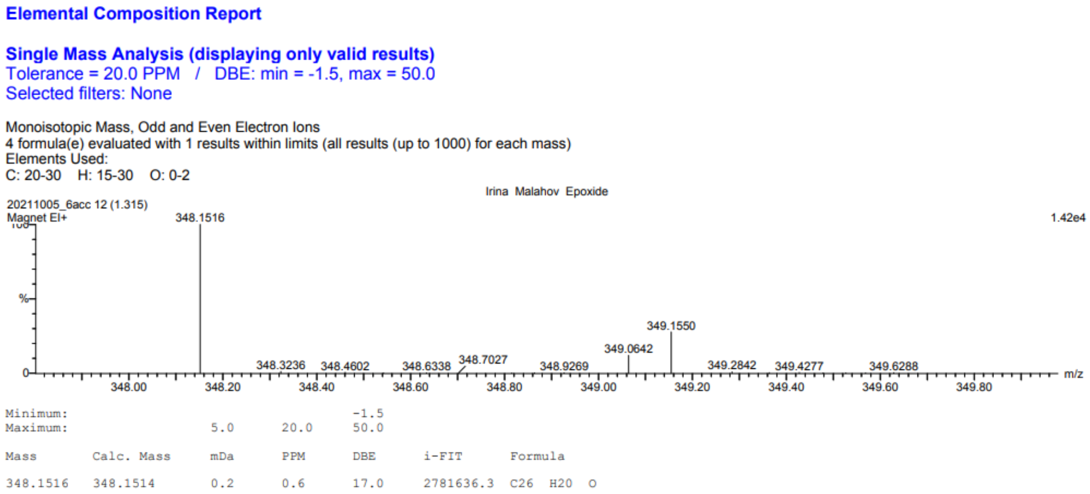

Figure S18. HRMS spectrum of **4.**

1-OK:

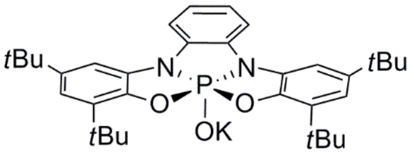

^{1}H NMR (400 MHz, d8-toluene); *δ* = 7.46 (s, 2H, Ar-*H*), 7.21 (s, 2H, Ar-*H*), 7.04 (s, 2H, Ar-*H*), 6.78 (s, 2H, Ar-*H*), 1.53 (s, 18H, -C(CH_3)$_3$), 1.36 (s, 18H, -C(CH_3)$_3$) ppm. **^{13}C NMR (100 MHz, d8-toluene); *δ*** = 142.78, 140.09, 132.37 (d, *J* = 9 Hz), 131.68 (d, *J* = 19 Hz), 120.69, 114.59, 112.78 (d, *J* = 10 Hz), 104.24 (d, *J* = 10 Hz), 104.82 (d, *J* = 10 Hz), 35.15 (-*C*(CH_3)$_3$), 34.52 (-*C*(CH_3)$_3$), 32.31 (-C(*C*H_3)$_3$), 29.84 (-C(*C*H_3)$_3$) ppm. **^{31}P NMR (162 MHz, d8-toluene); *δ*** = -28.21 ppm. **EA**: Calc'd for $C_{34}H_{44}KN_2O_3P\cdot(3H_2O+O)$: C, 61.06; H, 7.54; N, 4.19; Obs: C, 61.04; H, 7.56; N, 4.21. Only partially hydrolyzed **1-OK** could be obtained after multiple attempts.

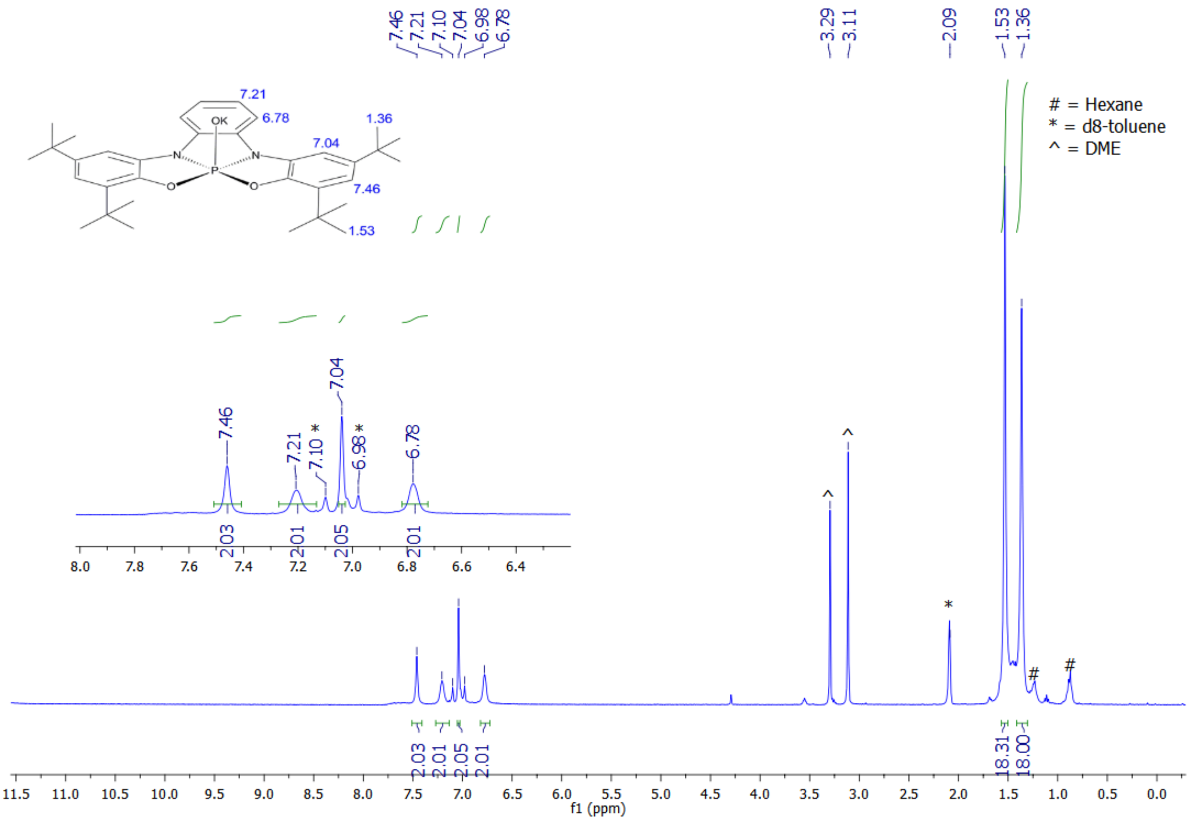

Figure S19. ^{1}H NMR spectrum (400 MHz, d8-toluene) of **1-OK.**

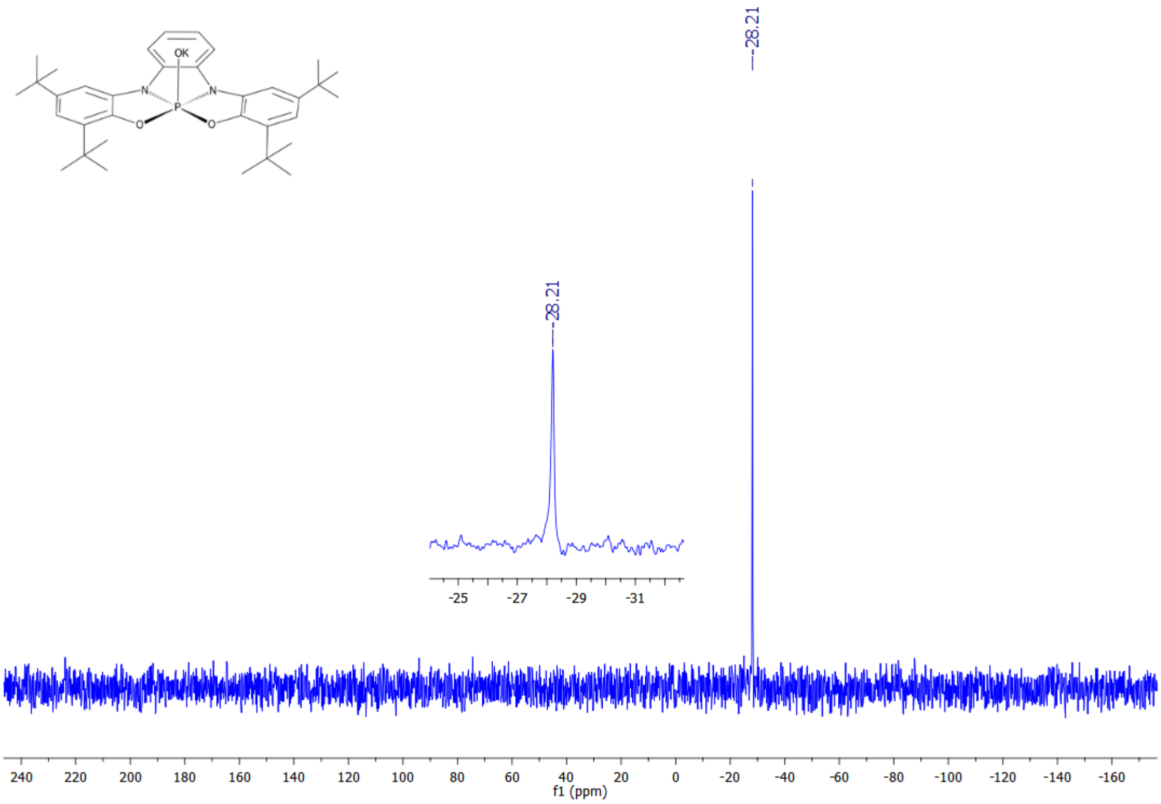

Figure S20. ^{31}P NMR spectrum (162 MHz, d8-toluene) of **1-OK.**

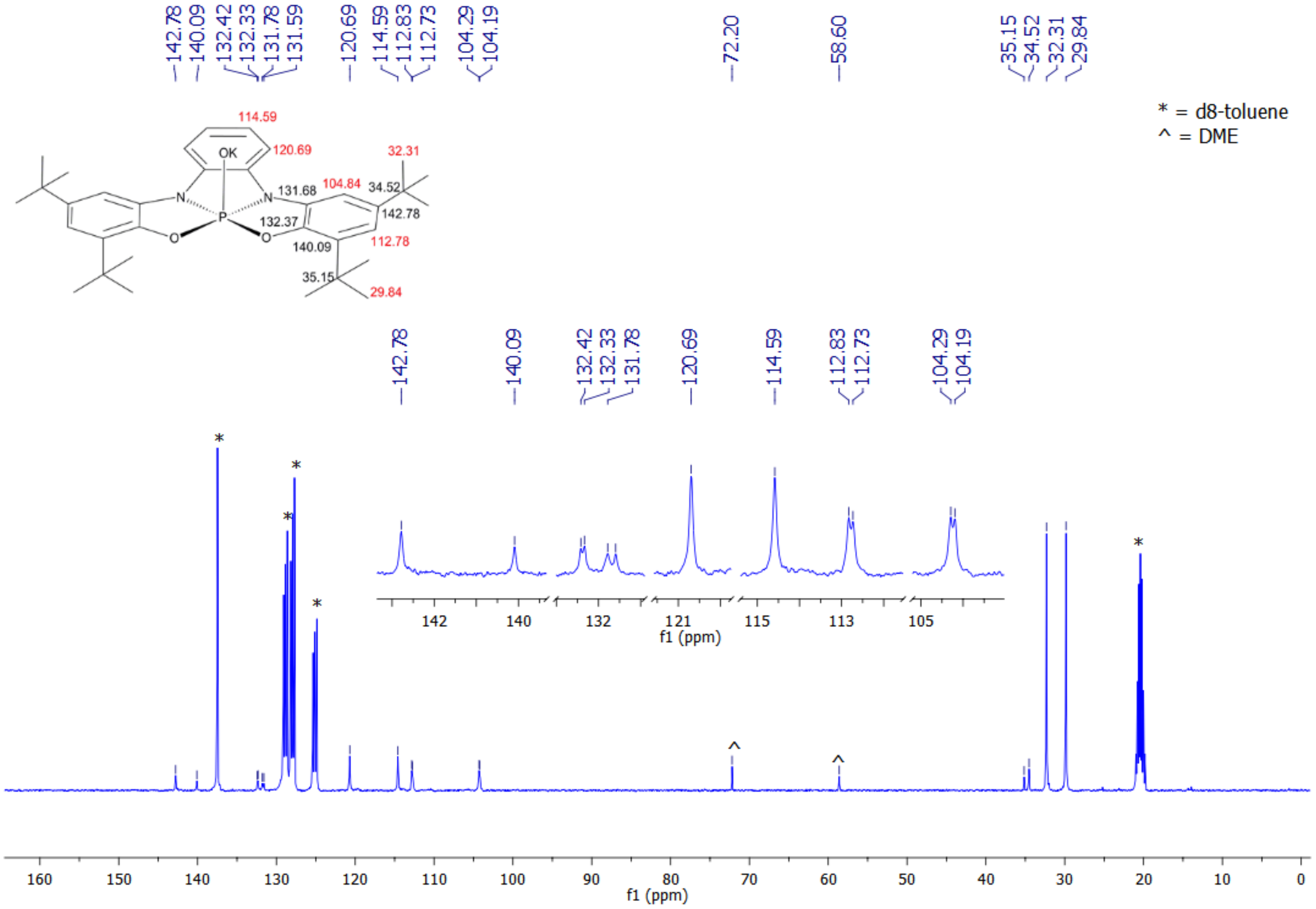

Figure S21. ^{13}C NMR spectrum (100 MHz, d8-toluene) of **1-OK.**

1-OH:

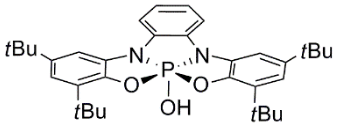

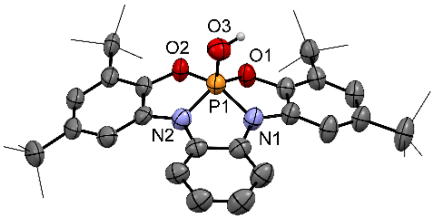

Molecular structure of **1-OH**

Yield: 130 mg (74.3%). **^{1}H NMR (400 MHz, $CDCl_3$); *δ*** = 7.24 (m, 3H, Ar-*H*), 7.14 (m, 1H, Ar-*H*), 6.98 (br, 1H, Ar-*H*), 6.86 (br, 2H, Ar-*H*), 6.69 (s, 1H, Ar-*H*), 1.45 (s, 18H, -C(CH_3)$_3$), 1.20 (s, 18H, -C(CH_3)$_3$) ppm. **^{13}C NMR (100 MHz, $CDCl_3$); *δ*** = 142.48, 139.47, 132.05, 131.05 (d, J = 18 Hz), 129.02 (d, J = 14 Hz), 128.36, 127.69, 127.19, 120.25, 114.25, 112.42, 103.56, 35.01 (-*C*(CH_3)$_3$), 34.22 (-*C*(CH_3)$_3$), 32.19 (-C(CH_3)$_3$), 29.53 (-C(CH_3)$_3$) ppm. **^{31}P NMR (162 MHz, $CDCl_3$); *δ*** = -28.22 ppm. **HRMS** (AP$^+$): Calculated for $C_{34}H_{46}N_2O_3P$: 561.3246 [M+H]$^+$; Obs: 561.3242. **EA:** Due to low reproducibility of this compound good EA was not obtained.

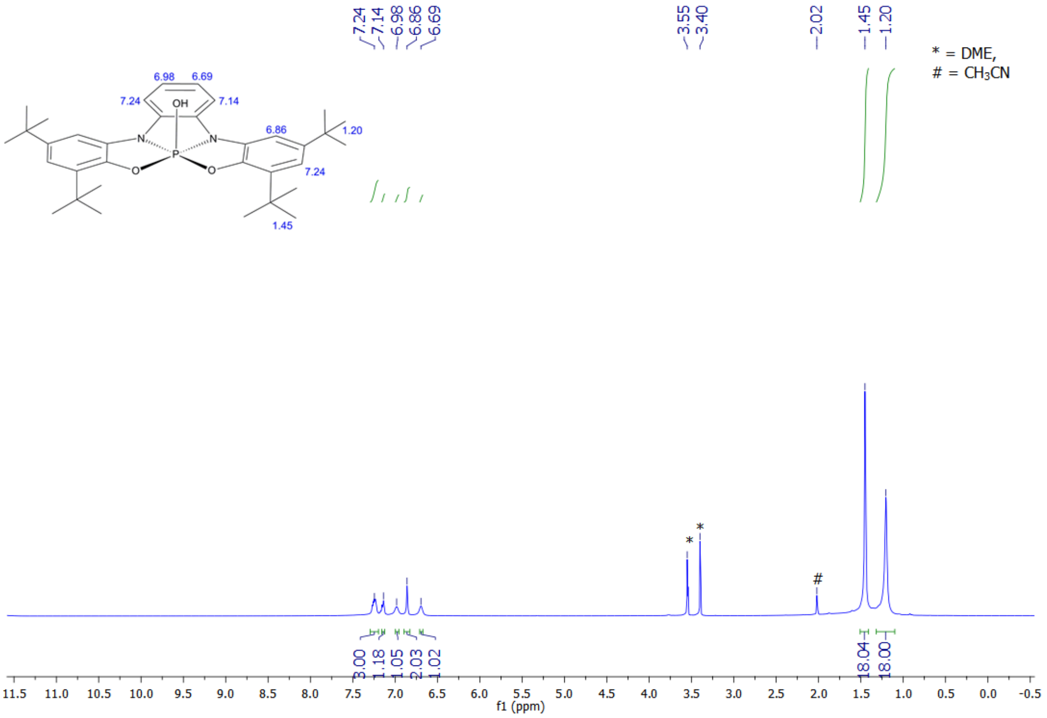

Figure S22. ^{1}H NMR spectrum (400 MHz, $CDCl_3$) of **1-OH.**

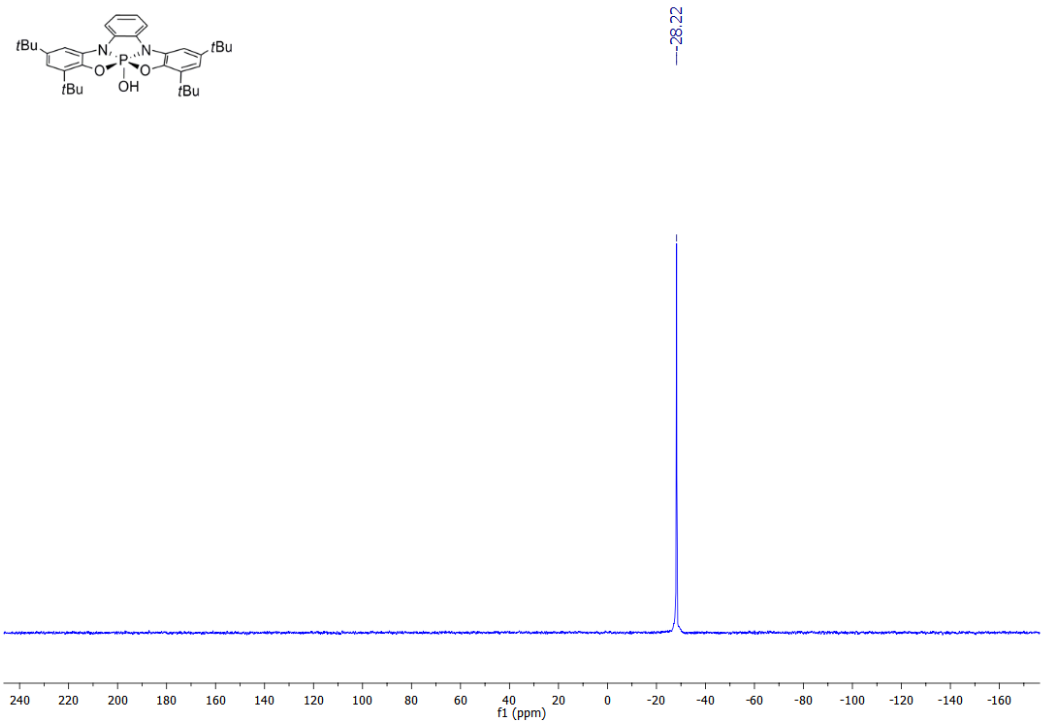

Figure S23. ^{31}P NMR spectrum (162 MHz, $CDCl_3$) of **1-OH.**

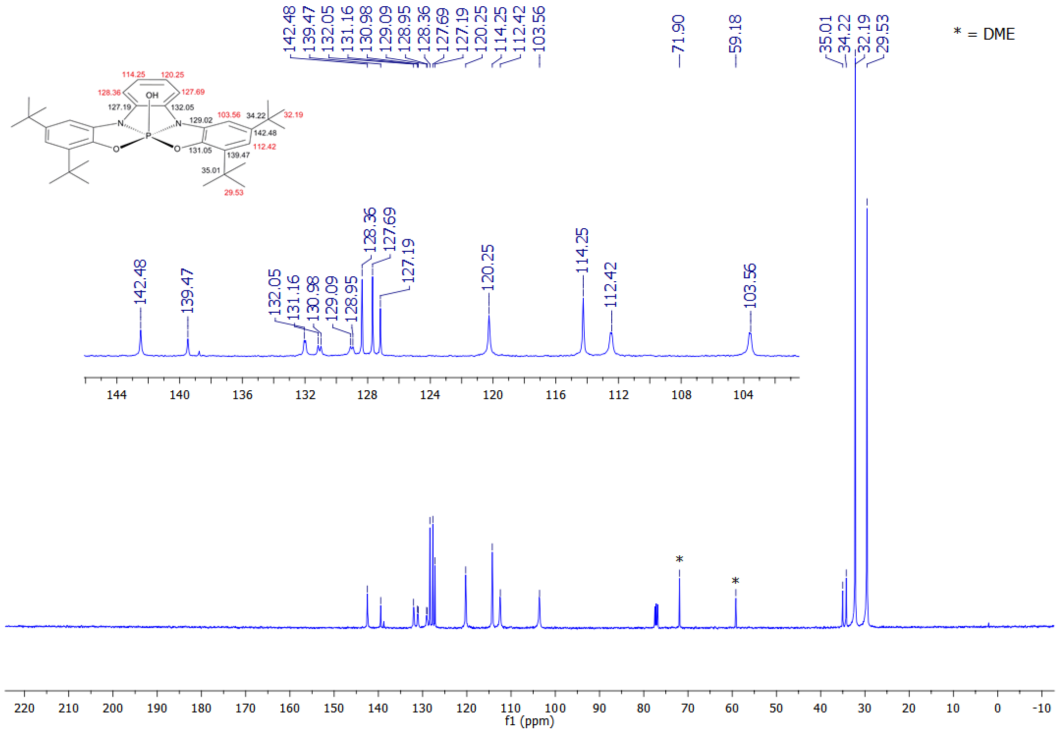

Figure S24. ^{13}C NMR spectrum (100 MHz, $CDCl_3$) of **1-OH.**

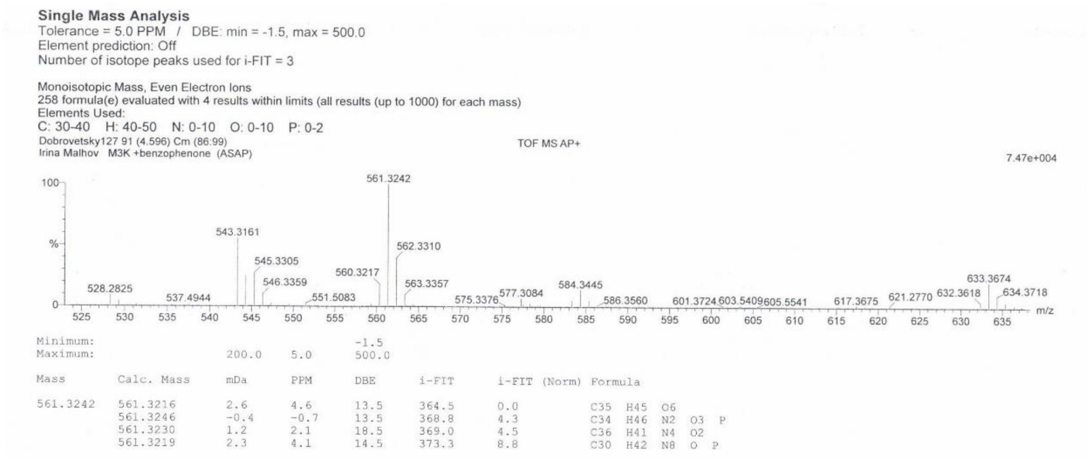

Mass	Calc. Mass	mDa	PPM	DBE	i-FIT	i-FIT (Norm)	Formula
561.3242	561.3216	2.6	4.6	13.5	364.5	0.0	C35 H45 O6
	561.3246	-0.4	-0.7	13.5	368.8	4.3	C34 H46 N2 O3 P
	561.3230	1.2	2.1	18.5	369.0	4.5	C36 H41 N4 O2
	561.3219	2.3	4.1	14.5	373.3	8.8	C30 H42 N8 O P

Figure S25. HRMS spectrum of **1-OH.**

Synthesis of 1-Me:

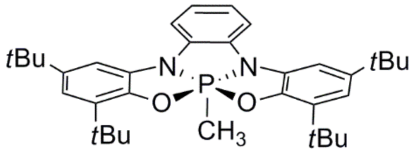

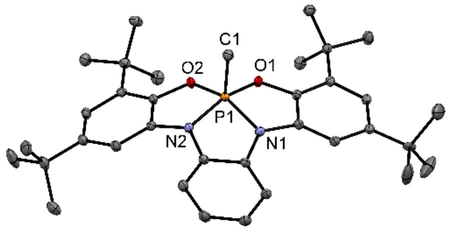

Molecular structure of **4**

Methyl iodide (22 µL, 0.353 mmol) was added to a 10 mL Et_2O solution of [**1**$^-$][K(18-crown-6)] (0.280 g, 0.330 mmol) at -30 °C. The reaction was stirred for 1h. The solution was filtered and all the volatiles were evaporated under vacuum. The obtained solid was dissolved in hexane kept for crystallization at room temperature after 2 weeks affording colorless crystals. Yield: 100 mg (54.25%).

Alternative path: The formation of **1-Me** (yield: 110 mg, 59.58%) could also be observed on performing the reaction in a DME solution through in situ generation of **1**$^-$ using KHMDS and without 18-crown-6.

^{1}H NMR (400 MHz, $CDCl_3$); *δ* = 7.61 (m, 2H, Ar-*H*), 7.52 (s, 2H, Ar-*H*), 7.08 (m, 2H, Ar-*H*), 7.01 (s, 2H, Ar-*H*) 1.50 (s, 18H, -C(CH_3)$_3$), 1.42 (s, 18H, -C(CH_3)$_3$), 0.01 (s, 3H, P-CH_3) ppm. **^{13}C NMR (100 MHz, $CDCl_3$); *δ*** = 143.58, 139.35, 133.20 (d, J = 9 Hz), 129.99 (d, J = 13 Hz), 129.74 (d, J = 19 Hz), 120.50, 115.55, 110.16 (d, J = 10 Hz), 106.97 (d, J = 12 Hz), 35.00 (-*C*(CH_3)$_3$), 34.51 (-*C*(CH_3)$_3$), 31.88 (-C(*C*H_3)$_3$), 29.47 (-C(*C*H_3)$_3$), 18.47 (d, $J_{C\text{-}P}$ = 274 Hz, P-*C*H_3) ppm. **^{31}P NMR (162 MHz, $CDCl_3$); *δ*** = -18.14 (m) ppm. **HRMS** (AP$^+$): Calculated for $C_{35}H_{48}N_2O_3P$: 559.3453 [M+H]$^+$; Obs: 559.3464. **EA**: Calc'd for $C_{35}H_{47}N_2O_2P\cdot0.025(CH_3CN)$: C, 75.21; H, 8.48; N, 5.07; Obs: C, 75.20; H, 8.46; N, 5.07.

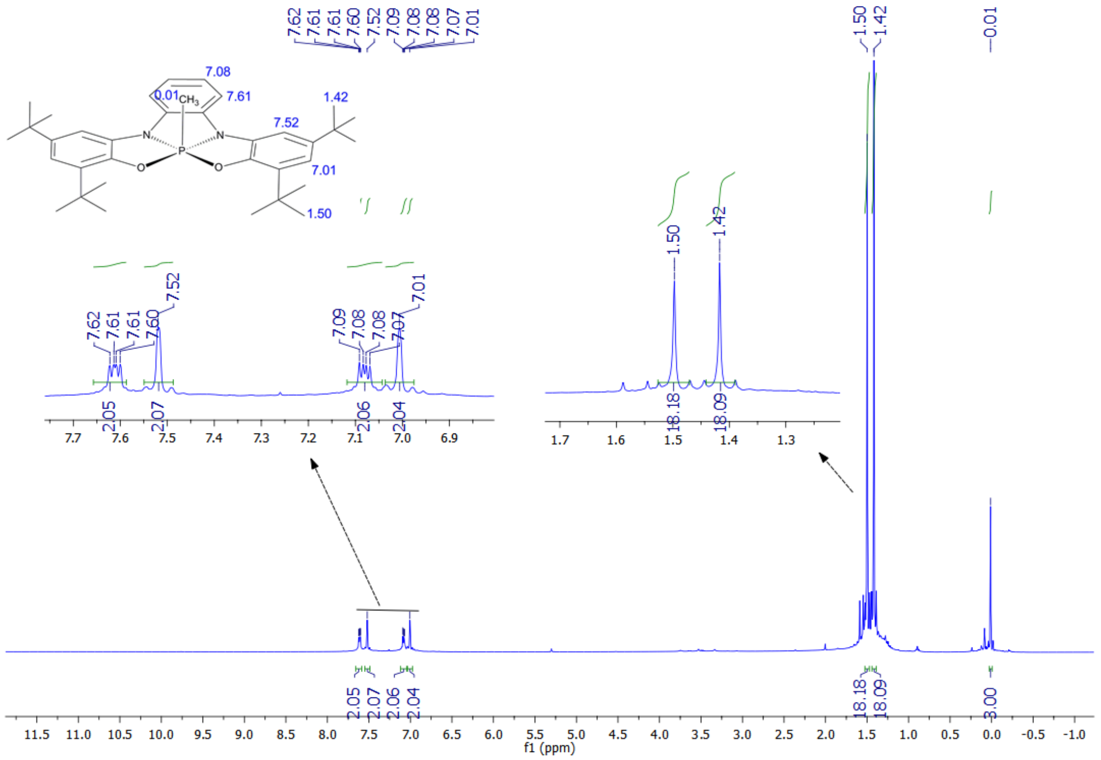

Figure S26. ^{1}H NMR spectrum (400 MHz, $CDCl_3$) of **1-Me.**

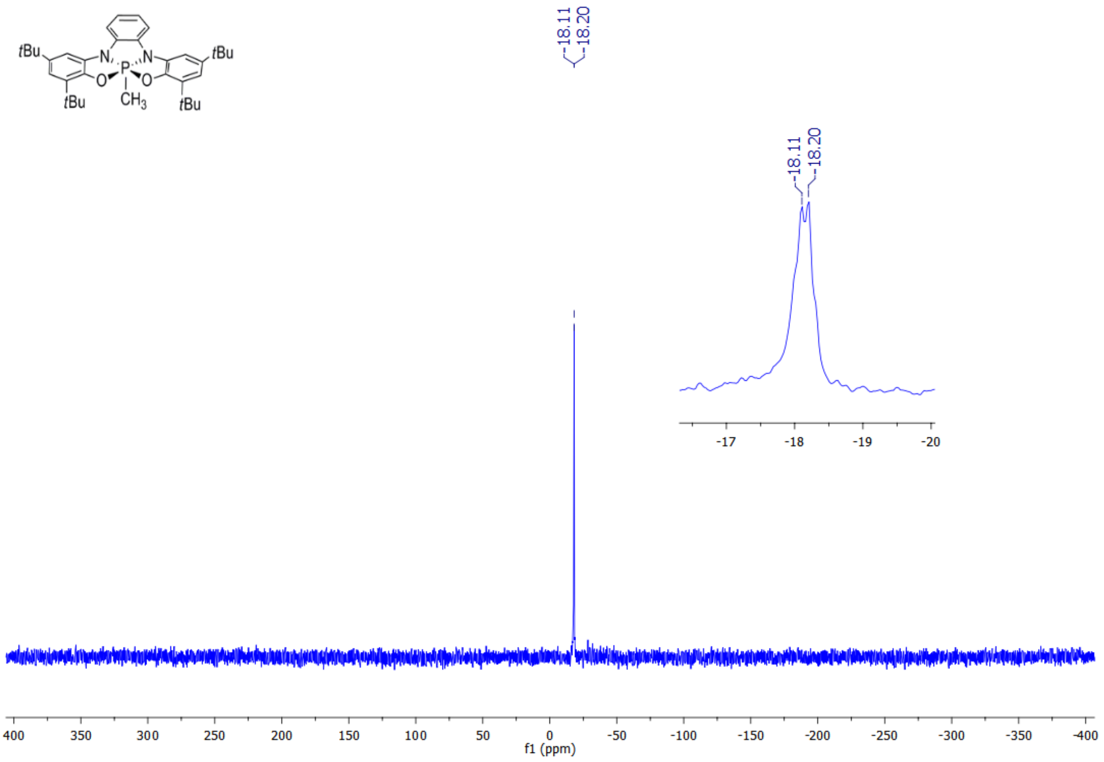

Figure S27. ^{31}P NMR spectrum (162 MHz, $CDCl_3$) of **1-Me.**

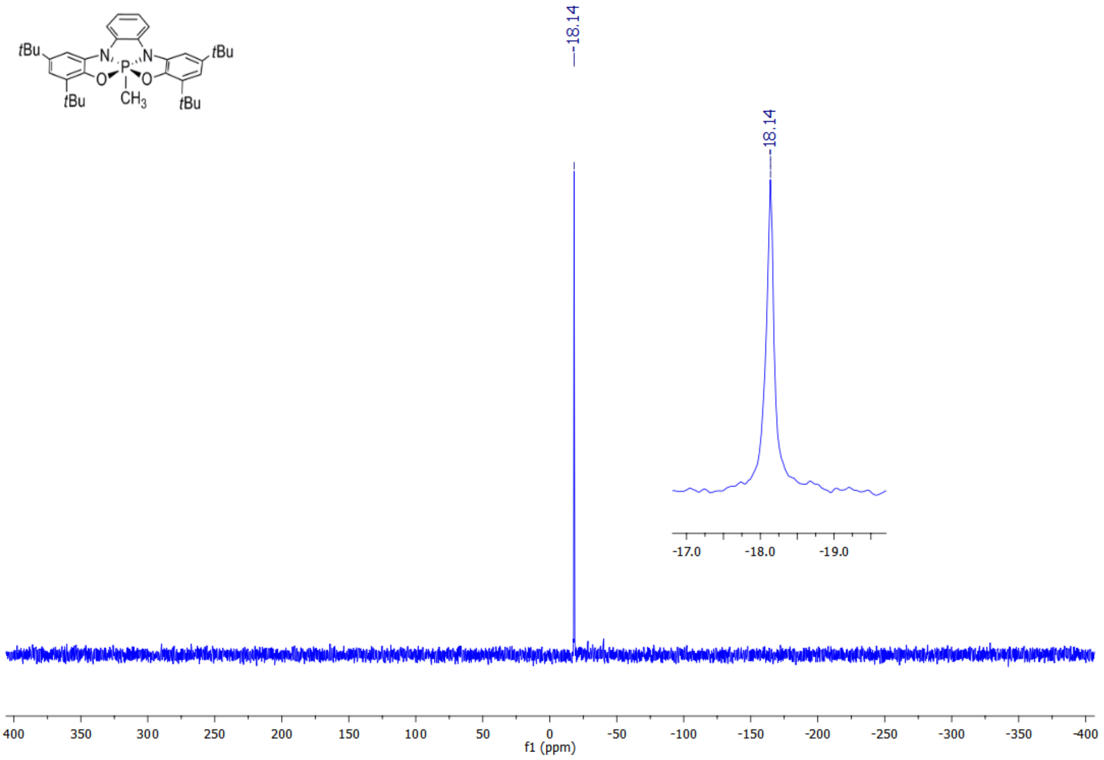

Figure S28. ^{31}P{^{1}H} NMR spectrum (162 MHz, $CDCl_3$) of **1-Me.**

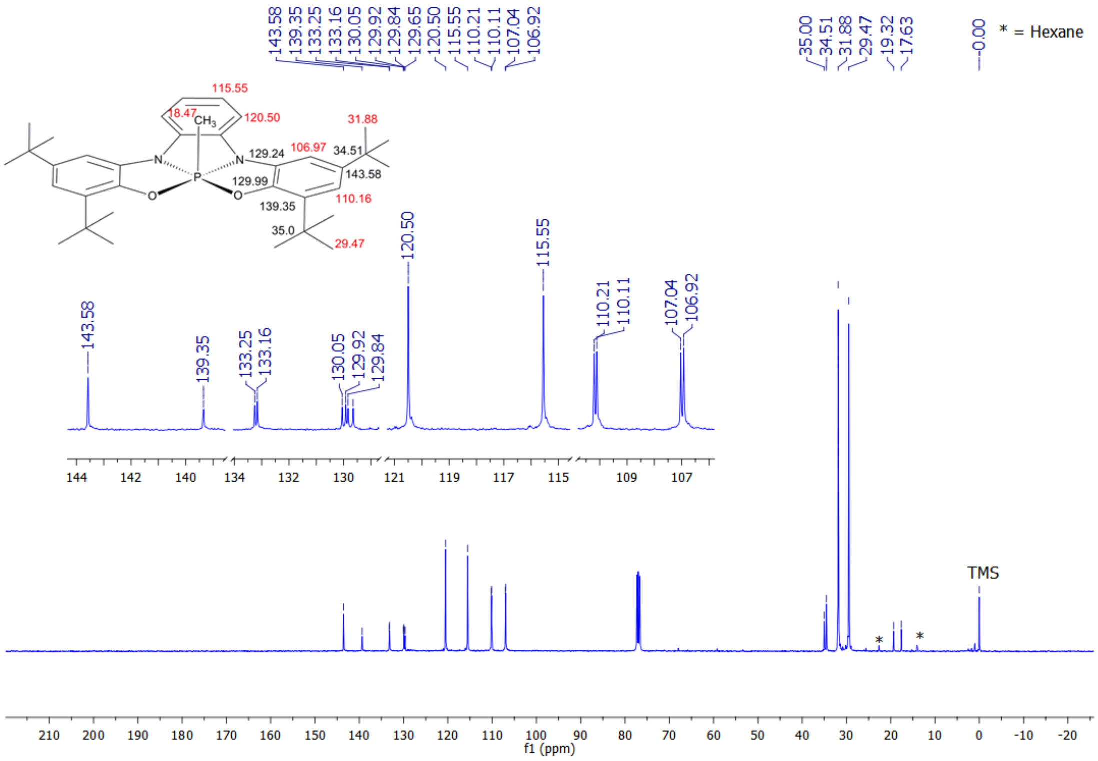

Figure S29. ^{13}C NMR spectrum (100 MHz, $CDCl_3$) of **1-Me.**

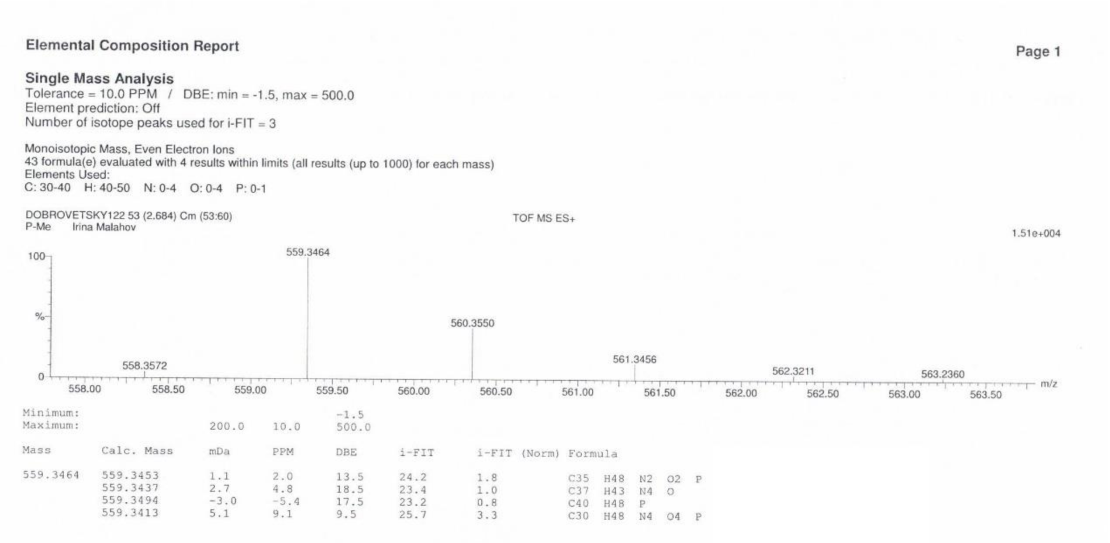

Minimum:				-1.5			
Maximum:		200.0	10.0	500.0			
Mass	Calc. Mass	mDa	PPM	DBE	i-FIT	i-FIT (Norm)	Formula
559.3464	559.3453	1.1	2.0	13.5	24.2	1.8	C35 H48 N2 O2 P
	559.3437	2.7	4.8	18.5	23.4	1.0	C37 H43 N4 O
	559.3494	-3.0	-5.4	17.5	23.2	0.8	C40 H48 P
	559.3413	5.1	9.1	9.5	25.7	3.3	C30 H48 N4 O4 P

Figure S30. HRMS spectrum of **1-Me.**

4. Electrochemistry:

The electrochemical measurements were carried out using a CHI760E electrochemical workstation under an inert atmosphere in a glovebox. A 3 mm glassy carbon served as the working electrode, Ag/Ag$^+$ was used as the reference electrode, and a Pt wire as the counter electrode. All electrodes were rinsed with the anhydrous THF prior to use. [nBu$_4$N][ClO$_4$] (0.1 M) in anhydrous THF was used as the supporting electrolyte. Cyclic voltammetry (CV) was performed at a scan rate of 0.1 V/s.

Calculation of p*K*a: 0.8 mL THF solution of **1-H** (58.7 mg, 0.1077 mmol) was added to an equimolar amount of 9-fluorenyl potassium (22 mg, 0.1077 mmol) at room temperature. ^{31}P NMR spectrum of this mixture showed a equilibrium between **1-H** (30 %) and **1**$^{-}$ (70 %). Based on this equilibrium the p*K*a value for **1-H** in THF was calculated as 23.67.[16]

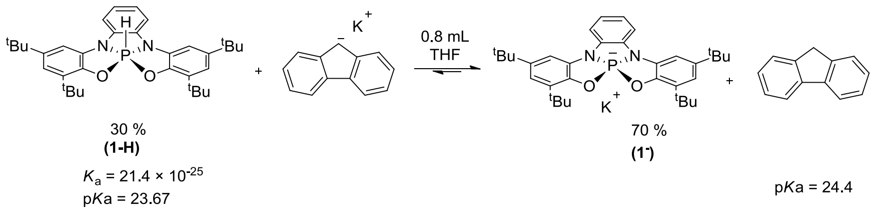

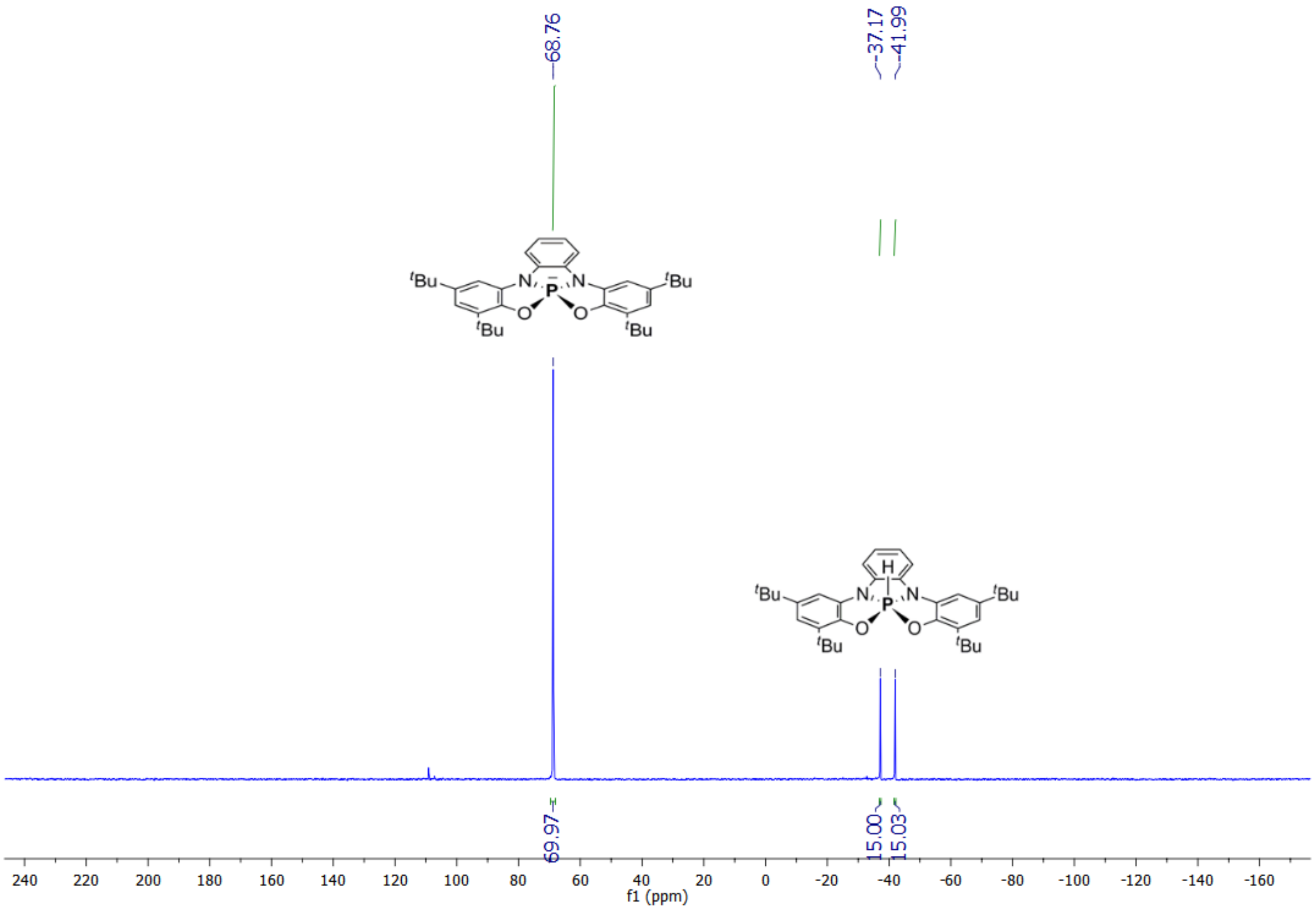

Figure S31. ^{31}P NMR spectrum (162 MHz, $CDCl_3$) to show the equilibrium of **1H** and **1**$^{-}$.

Bond Dissociation Free Energies (BDFE) for 1-H: BDFE(P-H) for **1-H** was calculated as 111.1775 Kcalmol^{-1} using Bordwell equation.[17]

$$\mathrm{BDFE_{sol}(X\text{-}H)\,)} = 1.37\mathrm{p}K_a + 23.06E° + C_{G,sol}$$

The values for $E°$ and $C_{G,sol}$ were used as 1.16 (with respect to ferrocene (0.41))[18] and 52, respectively.[19]

5. EPR measurements:

The EPR spectra were recorded on a Bruker EMX-10/12 X-band (v= 9.3 GHz) digital EPR spectrometer equipped with a Bruker N2-temperature controller. The spectra were recorded at a microwave power of

1-50 mW, 100 kHz magnetic field modulation of 0.1-2.0 G amplitude. Digital field resolution was 2048 points per spectrum. This allowed all hyperfine splitting to be measured directly with accuracy better than 0.1 G. Spectra processing and simulation were performed with the Bruker WIN-EPR and SimFonia Software. The g-factor values were determined using 2,2,6,6-tetramethylpiperidine-N-oxyl (TEMPO) as a reference (g =2.0058). When the reactions were carried out under UV irradiation, a high-pressure mercury lamp (1 kW) (ARC lamp power supply model 69920) was used, with the output being focused onto the sample with a quartz lens and filtered through distilled water to remove infrared radiation.

Generation of $1^•$ radical through $[Ph_3C][B(C_6F_5)_4]$:

$[Ph_3C][B(C_6F_5)_4]$ (95 mg, 0.1 mmol) was added to 0.6 mL E_2O solution of [**1**$^-$][K(18-crown-6)] (85 mg, 0.1 mmol) at room temperature. A quick EPR measurement showed the formation of Ph_3C• radical. The ^{31}P NMR spectrum confirmed the formation of **1-1**. All the volatiles were evaporated in vacuum and the mixture was extracted with 5 mL hexane and few drops of acetonitrile were added. The slow evaporation of this solution at room temperature afforded colorless crystals. Yield: 40 mg (37%).

Generation of $1^•$ radical through tert-butyl peroxide:

Tert-butyl peroxide (0.8 mL, 4.35 mmol) was added to a J Young NMR tube containing 0.2 mL benzene solution of **1-H** (10 mg, 0.018 mmol). After degassing by a 5-fold freeze-thaw procedure the EPR was recorded under UV-irradiation at 330 K temperature.

Generation of $[1\text{-}OCPh_2]^•$ radical:

1-H (10 mg, 0.0184 mmol) and benzophenone (15 mg, 0.0823 mmol) were added to a J Young NMR tube containing 0.8 mL tert-butyl benzene and 0.2 ml (1.0 mmol) tert-butyl peroxide. After degassing by a 3-fold freeze-thaw procedure the sample was UV-irradiated at 300 K (20 min) and observed at 370K temperature without UV-irradiation.

Generation of $[1\text{-}OC(C_6F_5)_2]^•$ radical:

KHMDS (9 mg, 0.0451 mmol) was added to a J Young NMR tube containing 1 mL DME solution of compound **1-H** (23 mg, 0.0442 mmol) at room temperature, followed by the addition of excess decafluorobenzophenone. The EPR was recorded under room temperature without UV-irradiation.

Reaction in presence of C_{60}:

To support the formation of **1**$^•$ radical, the radical trap C_{60} was used. Into a 25 mL flask, C_{60} (22 mg, 0.03 mmol) and tert-butyl benzene (10 mL) were added. Argon was bubbled through the mixture via a capillary for 10 min, the flask was plugged with a glass stopper, and the content was stirred with a magnetic bar for 3 h. To the obtained 1 mL magenta fullerene solution were added **1-H** (10 mg, 0.018 mmol) and one drop of tert-butyl benzene. The solution was transferred into a J. Young NMR tube. After degassing by a 5-fold freeze-thaw procedure the EPR was recorded under UV-irradiation.

An EPR spectrum exhibited a doublet of quintet spectrum [$a_P(^{31}P)$ = 86.9 G, $a_N(2^{14}N)$ = 0.49 G, g = 2.003] (Figure 28). We also managed to observe two pairs of satellite lines from the interaction of the unpaired electron with the ^{13}C carbons of the fullerene core [$a(4^{13}C)$ = 3.4 G; $a(2^{13}C)$ = 8.8 G]. DFT calculations of the structure, spin density and EPR parameters[10-15] of **1-C$_{60}^•$** ([$a(^{31}P)$ = 97.0 G, $a(2^{14}N)$ = 0.24 G, g = 2.002]), agree with the experimental data. Importantly, when the irradiation was stopped, the EPR signal dropped significantly and totally disappeared after ~1 min. It is important to note that C_{60} radical adducts tend to dimerize through formation of C−C bonds, however, these dimers upon irradiation dissociate back to C_{60} based radicals.[7-9] In contrast, continuous irradiation of our reaction mixture did not reproduce the signal associated with **1-C$_{60}^•$** adduct. In addition, NMR and MS analysis of the reaction mixture had significant amounts of **1-1** dimer. This means that **1-C$_{60}^•$** probably dissociates back to C_{60} and **1**$^•$, which dimerizes, making this process irreversible (Figure 28).[7-9]

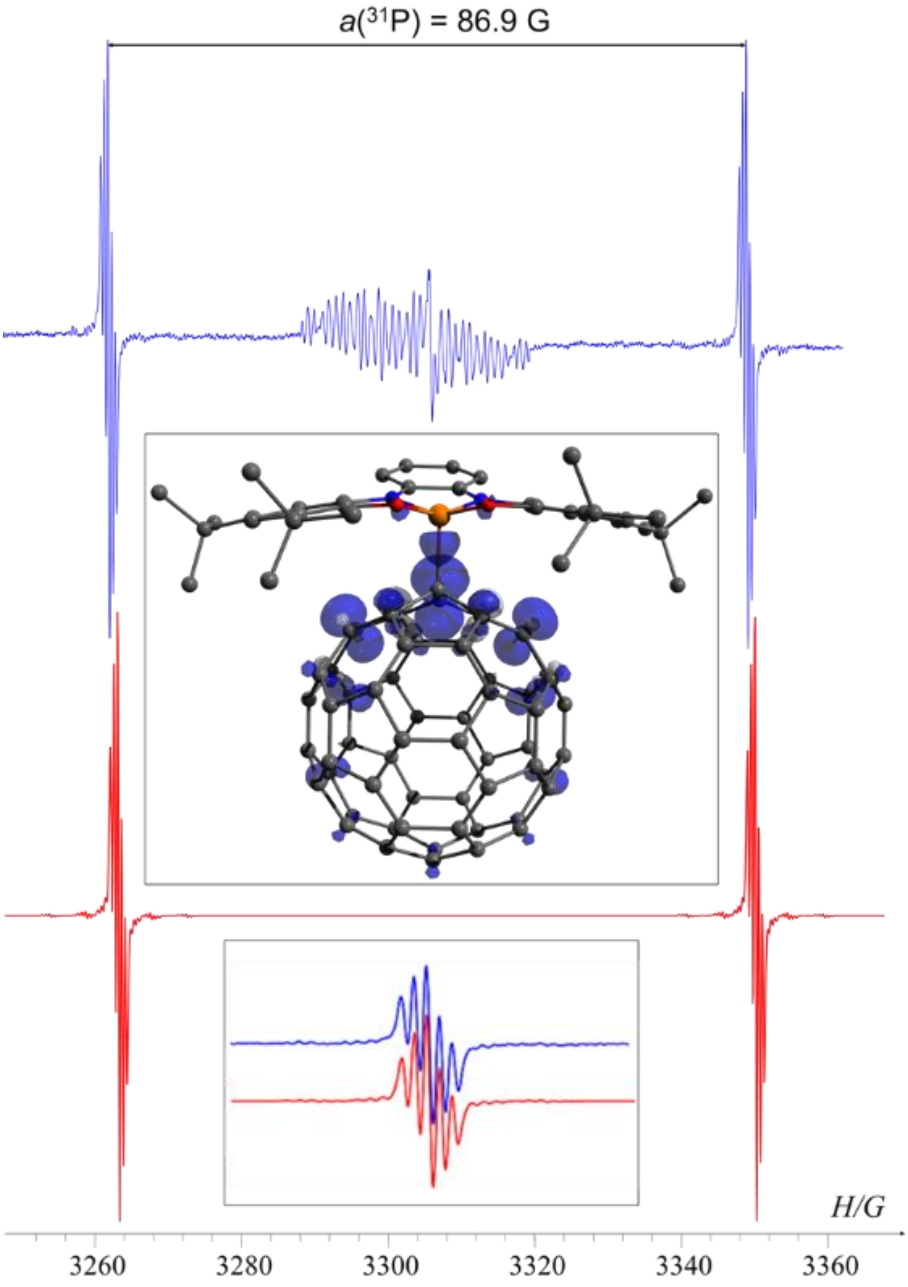

Figure S32. EPR spectra of **1-C$_{60}$$^{•}$** under UV-irradiation (λ > 300nm) (blue) and its simulation (red); in the top inset the DFT calculated Mulliken atomic spin densities of **1-C$_{60}$$^{•}$**. in the bottom inset expanded multiplet of the low-field component of the doublet (blue) and its simulation (red).

Analysis Info

Analysis Name	D:\Data\Apeloig\Ap_9037 .d	Acquisition Date	08/08/2021 12:18:38
Method	APCI_pos_SolidProbe.m	Operator	Larisa Panz
Sample Name	BENFP	Instrument	maXis impact
Comment			

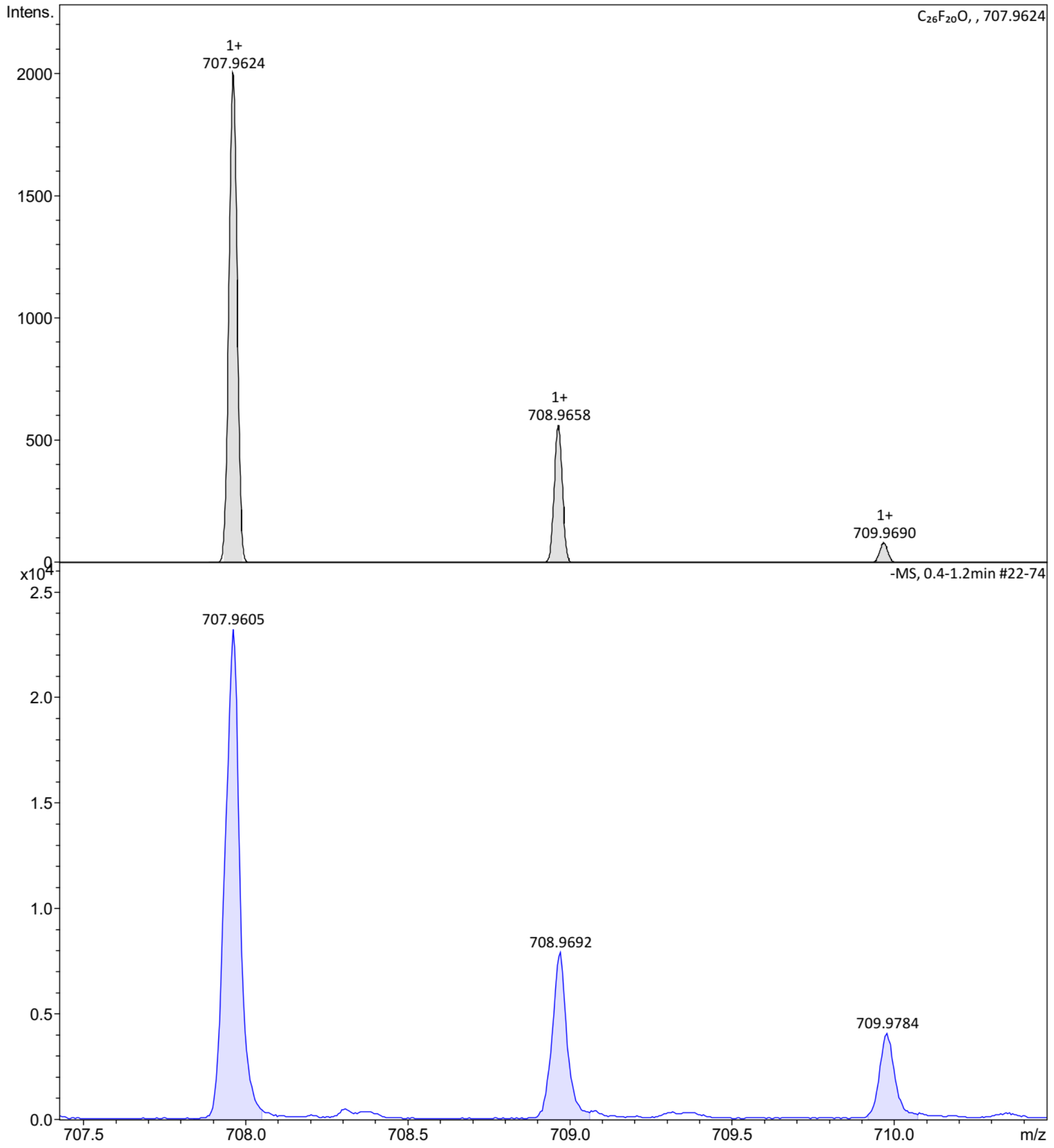

Bruker Compass DataAnalysis 4.2 printed: 08/08/2021 12:30:51 by: Larisa Panz Page 1 of 1

Figure S33. HRMS spectrum (APCI$^+$) of tetrakis-(C_6F_5)-epoxide. *m/z* calculated for $C_{26}F_{20}O$ 707.9624 $[M]^+$; obs: 707.9605.

6. DFT Computations

DFT calculations were performed using Gaussian 09.2.[10] Geometry optimizations of **1**$^{\bullet}$ and **[1-OCPh$_2$]**$^{\bullet}$ were optimized using the uwB97XD/def2-TZVP level of theory,[11,12] **[1-OC(C$_6$F$_5$)$_2$]**$^{\bullet}$ was optimized using uB3LYP-D3/def2-SVP level of theory,[13-14] implemented in the Gaussian 09 software. Thermal energy corrections were extracted from the results of frequency analysis performed at the same level of theory. Frequency analysis of all the molecules and intermediates contained no imaginary frequency showing that these are energy minima.

Optimized geometries

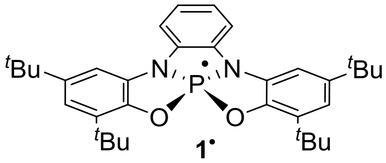

1$^{\bullet}$:

N	-1.21403	1.19857	-0.53189
N	1.16608	1.16086	-0.37044
P	0.00473	-0.03324	-0.88128
C	-2.46537	0.66385	-0.27849
C	2.44591	0.65417	-0.16725
C	2.42086	-0.72631	-0.33256
C	3.55852	-1.51521	-0.18896
C	4.72106	-0.83025	0.14654
C	4.77996	0.54981	0.34715
C	3.62150	1.29753	0.18852
C	-2.34260	-0.72059	-0.12524
C	-3.43292	-1.52935	0.15819
C	-4.65015	-0.85698	0.29102
C	-4.81169	0.51589	0.15103
C	-3.69306	1.28644	-0.14804
H	5.63174	-1.39828	0.26781
C	3.50479	-3.03129	-0.37964
H	-5.52602	-1.44956	0.51956
C	-3.41030	-3.05232	0.34912
O	-1.06293	-1.15206	-0.25930
O	1.19186	-1.20701	-0.60913
C	-2.03601	-3.70007	0.14658
C	-4.37962	-3.69222	-0.65841
C	-3.87279	-3.37516	1.77951
H	-4.07396	-3.47157	-1.68282
H	-5.40243	-3.33679	-0.52882
H	-4.38517	-4.77698	-0.53035
H	-1.64275	-3.52476	-0.85385
H	-2.13898	-4.77848	0.28691
H	-1.30136	-3.33560	0.86272
C	3.04148	-3.35195	-1.81013
C	2.52497	-3.64420	0.63487
C	4.87394	-3.68200	-0.16962
H	2.05138	-2.94598	-2.00928
H	3.00120	-4.43421	-1.95459
H	3.73888	-2.93785	-2.54146
H	5.61422	-3.31301	-0.88262
H	4.78223	-4.75939	-0.31771
H	5.25336	-3.51848	0.84115
H	-3.19611	-2.93151	2.51221
H	-3.88091	-4.45656	1.93437
H	-4.87748	-3.00033	1.97861
H	1.51669	-3.25606	0.50223
H	2.84614	-3.43118	1.65673
H	2.49194	-4.72895	0.50852
C	-0.72092	2.47887	-0.44480
C	-1.40176	3.68836	-0.46449
C	0.68653	2.45919	-0.34993
C	-0.68431	4.87089	-0.37777
H	-2.47431	3.72162	-0.56607

C	1.39356	3.65191	-0.29735
C	0.69952	4.85229	-0.30060
H	-1.21301	5.81485	-0.38969
H	2.47033	3.66457	-0.27561
H	1.25097	5.78215	-0.25649
H	-3.78142	2.35081	-0.27144
H	3.63424	2.35744	0.36432
C	-6.20035	1.13530	0.33180
C	6.11351	1.19579	0.73501
C	7.14888	0.93473	-0.36960
H	8.10581	1.39264	-0.10799
H	7.31867	-0.13204	-0.52012
H	6.81503	1.35838	-1.31881
C	5.99263	2.71018	0.92134
H	5.67249	3.20581	0.00226
H	5.29018	2.96705	1.71700
H	6.96504	3.12355	1.19474
C	6.61218	0.59110	2.05643
H	5.88609	0.75591	2.85485
H	6.77727	-0.48353	1.97007
H	7.55759	1.05271	2.35093
C	-6.18847	2.65285	0.13163
H	-5.53571	3.15151	0.85123
H	-5.86706	2.92487	-0.87611
H	-7.19595	3.04796	0.27342
C	-7.17350	0.52775	-0.68972
H	-7.27391	-0.55058	-0.56015
H	-8.16547	0.97135	-0.57587
H	-6.83026	0.71260	-1.70940
C	-6.70460	0.84521	1.75386
H	-6.79918	-0.22595	1.93704
H	-6.01808	1.25313	2.49823
H	-7.68687	1.29845	1.90758

Sum of electronic and zero-point Energies= -1922.856034
Sum of electronic and thermal Energies= -1922.817873
Sum of electronic and thermal Enthalpies= -1922.816929
Sum of electronic and thermal Free Energies= -1922.924928

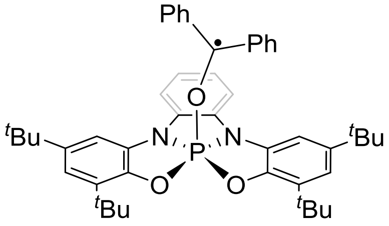

[1-OCPh$_2$]$^•$:

N	-1.22704	1.73207	-0.27664
N	1.12022	1.75604	0.05052
C	-2.52507	1.32283	0.01326
C	2.40077	1.31760	0.39985
C	2.30790	0.03264	0.91224
C	3.41185	-0.69002	1.33461
C	4.63642	-0.04648	1.17136
C	4.77743	1.22454	0.61905
C	3.63534	1.91698	0.22988
C	-2.46951	0.20706	0.83443
C	-3.59682	-0.39502	1.36723
C	-4.80683	0.14254	0.93317
C	-4.90950	1.21737	0.05016
C	-3.74464	1.82466	-0.40651
H	5.53070	-0.56869	1.47721
C	3.27628	-2.09508	1.92065
H	-5.71979	-0.30331	1.29885
C	-3.49778	-1.51332	2.40504
O	-1.18989	-0.20553	1.07138
O	1.03205	-0.43142	0.92131
C	-2.70913	-2.70915	1.85457

C	-4.88035	-2.01498	2.82833
C	-2.78607	-0.95803	3.65031
H	-5.43256	-2.43379	1.98427
H	-5.48257	-1.22460	3.28115
H	-4.76236	-2.80533	3.57159
H	-3.19946	-3.12389	0.97481
H	-2.65598	-3.49194	2.61512
H	-1.69273	-2.43644	1.57856
C	2.66516	-3.04071	0.87469
C	2.37406	-2.04607	3.16448
C	4.63272	-2.66671	2.34021
H	1.65320	-2.73921	0.60994
H	2.62323	-4.05577	1.27733
H	3.26518	-3.05612	-0.03762
H	5.30952	-2.77309	1.48988
H	4.48509	-3.65863	2.77076
H	5.11949	-2.04768	3.09682
H	-1.76944	-0.64144	3.41906
H	-2.73219	-1.72981	4.42164
H	-3.32923	-0.10384	4.05987
H	1.37357	-1.69151	2.92086
H	2.79842	-1.38475	3.92299
H	2.28337	-3.04515	3.59719
C	-0.72459	2.99364	-0.53189
C	-1.40209	4.14895	-0.87528
C	0.66648	3.01516	-0.33593
C	-0.68564	5.32953	-1.03253
H	-2.47180	4.14715	-1.01228
C	1.36817	4.19776	-0.47652
C	0.68221	5.35393	-0.83469
H	-1.21008	6.23520	-1.30739
H	2.42896	4.24903	-0.29628
H	1.23097	6.27874	-0.95324
H	-3.78443	2.64828	-1.09888
H	3.71716	2.88595	-0.22736
C	-6.29748	1.69252	-0.38986
C	6.17819	1.82169	0.45321
C	7.02673	0.90315	-0.43904
H	8.02545	1.32477	-0.57515
H	7.13935	-0.09080	-0.00450
H	6.56755	0.78882	-1.42314
C	6.14378	3.20971	-0.19134
H	5.70169	3.18216	-1.18960
H	5.58328	3.92315	0.41654
H	7.16094	3.59237	-0.29247
C	6.84338	1.95230	1.83184
H	6.24987	2.59288	2.48687
H	6.95209	0.98278	2.31974
H	7.83894	2.39147	1.73230
C	-6.22950	2.89341	-1.33634
H	-5.74745	3.75280	-0.86546
H	-5.69329	2.65588	-2.25742
H	-7.24115	3.19511	-1.61392
C	-7.01574	0.54780	-1.12065
H	-7.13396	-0.32686	-0.47977
H	-8.01056	0.86620	-1.44147
H	-6.45350	0.24269	-2.00577
C	-7.11606	2.10643	0.84264
H	-7.24787	1.27863	1.54020
H	-6.62204	2.91993	1.37755
H	-8.10839	2.44930	0.53996
P	-0.06302	0.52575	0.12859
O	-0.09004	-0.21475	-1.31108
C	0.13010	-1.54059	-1.59505
C	-1.04932	-2.38656	-1.52559
C	-0.98495	-3.74982	-1.20637
C	-2.31012	-1.83264	-1.79313
C	-2.12316	-4.53534	-1.22419
C	-3.44450	-2.62166	-1.80332
C	-3.35578	-3.98120	-1.53880
C	1.41057	-1.87512	-2.13451
C	2.44275	-0.91536	-2.15489

C	1.70514	-3.14521	-2.67489
C	3.71306	-1.23600	-2.58403
C	2.97728	-3.45383	-3.11028
C	3.99956	-2.51390	-3.04638
H	-2.39059	-0.77702	-2.01372
H	-4.40418	-2.16907	-2.01852
H	-4.24433	-4.59947	-1.55213
H	-2.04869	-5.58596	-0.97330
H	-0.04199	-4.18654	-0.90770
H	0.92624	-3.88642	-2.76988
H	2.24075	0.09208	-1.82950
H	4.48574	-0.47811	-2.55683
H	4.99860	-2.76694	-3.37703
H	3.17315	-4.43906	-3.51468

Sum of electronic and zero-point Energies= -2499.364054
Sum of electronic and thermal Energies= -2499.314913
Sum of electronic and thermal Enthalpies= -2499.313969
Sum of electronic and thermal Free Energies= -2499.444265

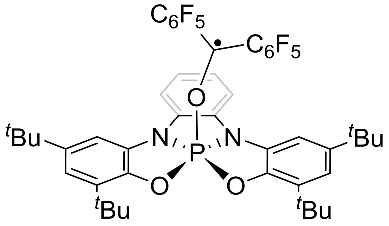

[1-OC(C$_6$F$_5$)$_2$]$^•$:

N	-1.59987	-1.91206	0.33722
N	0.65234	-2.09706	-0.37470
C	-2.92471	-1.53419	0.15042
C	1.79109	-1.85732	-1.14422
C	1.65945	-0.62119	-1.75710
C	2.55123	-0.15855	-2.71344
C	3.66150	-0.97281	-2.92566
C	3.85970	-2.19589	-2.28870
C	2.89040	-2.65575	-1.40467
C	-2.96215	-0.45543	-0.73015
C	-4.14358	0.10883	-1.17149
C	-5.30093	-0.45416	-0.62449
C	-5.30871	-1.51055	0.27515
C	-4.09096	-2.06552	0.66320
H	4.40287	-0.64494	-3.63877
C	2.27234	1.09418	-3.54604
H	-6.25077	-0.03245	-0.92438
C	-4.28590	1.25519	-2.18000
O	-1.70878	-0.07567	-1.12192
O	0.51093	0.01462	-1.39644
C	-2.95911	1.75066	-2.76656
C	-4.97568	2.44014	-1.48676
C	-5.15318	0.77445	-3.35543
H	-4.38655	2.78796	-0.63811
H	-5.96933	2.17464	-1.12304
H	-5.08563	3.26952	-2.18914
H	-2.28886	2.13822	-2.00273
H	-3.16883	2.55635	-3.47325
H	-2.43163	0.96093	-3.30082
C	2.04068	2.31120	-2.64473
C	1.00919	0.84175	-4.38728
C	3.42720	1.41870	-4.49600
H	1.15216	2.17431	-2.03334
H	1.89747	3.20649	-3.25426
H	2.89769	2.48121	-1.99026
H	4.35837	1.60287	-3.95605
H	3.18475	2.32354	-5.05599
H	3.59845	0.62104	-5.22133
H	-4.69345	-0.08402	-3.84884

H	-5.25781	1.57496	-4.09114
H	-6.15430	0.48258	-3.03656
H	0.14382	0.64684	-3.75384
H	1.15200	-0.01268	-5.05172
H	0.78866	1.71844	-5.00106
C	-1.04640	-3.08926	0.81294
C	-1.61343	-4.07133	1.60465
C	0.28945	-3.20167	0.39297
C	-0.84440	-5.16871	1.97386
H	-2.62771	-3.98837	1.95910
C	1.05481	-4.28314	0.78534
C	0.47470	-5.27074	1.57349
H	-1.28360	-5.93736	2.59599
H	2.09598	-4.36238	0.52100
H	1.07273	-6.11700	1.88452
H	-4.06216	-2.88818	1.35434
H	2.99028	-3.62189	-0.94589
C	-6.64522	-2.03363	0.81119
C	5.12321	-3.00612	-2.59541
C	6.35932	-2.17721	-2.21410
H	7.27311	-2.73603	-2.42990
H	6.40220	-1.23902	-2.76866
H	6.34886	-1.93690	-1.14911
C	5.17372	-4.32110	-1.81380
H	5.16210	-4.15146	-0.73498
H	4.34121	-4.97932	-2.07164
H	6.09729	-4.85151	-2.05268
C	5.17135	-3.33870	-4.09427
H	4.29672	-3.92151	-4.38971
H	5.19583	-2.43664	-4.70702
H	6.06617	-3.92210	-4.32390
C	-6.46228	-3.17865	1.81020
H	-5.96741	-4.03909	1.35479
H	-5.88422	-2.86610	2.68257
H	-7.43883	-3.51228	2.16579
C	-7.39025	-0.89488	1.52427
H	-7.61085	-0.06967	0.84620
H	-8.33821	-1.25851	1.92800
H	-6.79501	-0.50076	2.35010
C	-7.49737	-2.54966	-0.35814
H	-7.70209	-1.76397	-1.08668
H	-6.98820	-3.36380	-0.87781
H	-8.45582	-2.92497	0.00873
P	-0.47171	-0.81742	-0.36016
O	-0.23153	0.03170	1.02477
C	0.55192	1.08618	1.34054
C	0.34029	2.36932	0.70228
C	1.41765	3.21210	0.41547
C	-0.92932	2.85222	0.36912
C	1.25726	4.44053	-0.19058
C	-1.10842	4.07558	-0.24728
C	-0.01198	4.87232	-0.53578
C	1.42439	0.92033	2.48528
C	2.13744	-0.26154	2.72452
C	1.59079	1.93128	3.44397
C	2.94725	-0.43144	3.82976
C	2.40539	1.78383	4.54895
C	3.08755	0.59505	4.74862
F	-2.01349	2.17083	0.71298
F	-2.32691	4.51372	-0.52836
F	-0.17863	6.04049	-1.13007
F	2.31155	5.18763	-0.48081
F	2.65675	2.81781	0.68953
F	0.92278	3.07594	3.33634
F	2.51645	2.76387	5.43312
F	3.86528	0.44267	5.80549
F	3.61370	-1.56352	4.00026
F	2.09630	-1.25446	1.85045

Sum of electronic and zero-point Energies= -3491.910331
Sum of electronic and thermal Energies= -3491.851419
Sum of electronic and thermal Enthalpies= -3491.850475
Sum of electronic and thermal Free Energies= -3492.004978

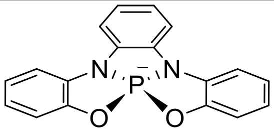

1m⁻
:

P	-0.03803	-1.03586	0.80332
O	0.99474	-1.88427	-0.33082
O	-1.32339	-2.10829	0.02231
N	1.25622	0.31768	0.70987
N	-1.13048	0.30268	0.31886
C	0.80950	1.55747	0.31389
C	1.54208	2.70916	0.07888
H	2.61174	2.71286	0.23010
C	0.89807	3.86660	-0.36072
H	1.47483	4.76905	-0.52520
C	-0.46273	3.86035	-0.58545
H	-0.96392	4.75552	-0.93363
C	-1.21012	2.70014	-0.36963
H	-2.26328	2.70155	-0.60041
C	-0.58708	1.55256	0.09264
C	2.47340	-0.22718	0.33335
C	2.27015	-1.48862	-0.25743
C	3.33581	-2.22430	-0.73639
C	4.62584	-1.70525	-0.61412
H	5.46822	-2.27901	-0.98241
C	4.83566	-0.47476	-0.01979
C	3.75715	0.27142	0.46083
H	3.92815	1.22137	0.94841
C	-2.44418	-0.10166	0.11775
C	-3.61800	0.62893	0.13313
H	-3.61159	1.68686	0.34780
C	-4.83774	-0.01779	-0.09175
C	-4.87366	-1.38001	-0.31714
H	-5.82193	-1.87489	-0.49315
C	-3.69852	-2.13231	-0.30035
C	-2.48770	-1.50209	-0.06403
H	-5.75475	0.55882	-0.08047
H	-3.71135	-3.20375	-0.45634
H	3.15473	-3.18412	-1.20305
H	5.84103	-0.08446	0.08341

Sum of electronic and zero-point Energies= -1294.321282
Sum of electronic and thermal Energies= -1294.304718
Sum of electronic and thermal Enthalpies= -1294.303774
Sum of electronic and thermal Free Energies= -1294.365666

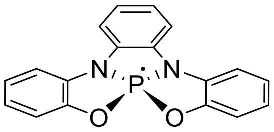

1m•
:

N	1.21303	0.35853	0.23060
N	-1.17092	0.34268	0.11955
P	-0.00178	-0.86831	0.61017
C	2.47653	-0.17151	0.03403
C	-2.45583	-0.15887	-0.04091
C	-2.40817	-1.55026	0.07497
C	-3.53754	-2.33298	-0.03285
C	-4.74957	-1.70176	-0.27825
C	-4.80969	-0.32307	-0.41297
C	-3.66875	0.46252	-0.29657
C	2.36294	-1.56137	-0.04808
C	3.45425	-2.37449	-0.25808
C	4.69881	-1.77232	-0.39505
C	4.82728	-0.39387	-0.31669
C	3.72269	0.42145	-0.09858
H	-5.65210	-2.29185	-0.36759

H	5.57379	-2.38636	-0.56252
O	1.10000	-2.01810	0.07898
O	-1.17424	-2.04036	0.27958
C	0.72659	1.64064	0.12133
C	1.42162	2.84120	0.08915
C	-0.68087	1.63329	0.05381
C	0.71566	4.02803	-0.02412
H	2.49623	2.86413	0.16657
C	-1.37609	2.83061	-0.03700
C	-0.66944	4.02229	-0.08415
H	1.25434	4.96591	-0.05219
H	-2.45253	2.85316	-0.05629
H	-1.21080	4.95612	-0.15761
H	3.85440	1.48948	-0.03782
H	-3.75052	1.52941	-0.41953
H	3.32537	-3.44634	-0.31954
H	5.80264	0.06206	-0.42375
H	-3.45853	-3.40663	0.06842
H	-5.75879	0.15750	-0.61005

Sum of electronic and zero-point Energies= -1294.235932
Sum of electronic and thermal Energies= -1294.219692
Sum of electronic and thermal Enthalpies= -1294.218748
Sum of electronic and thermal Free Energies= -1294.281494

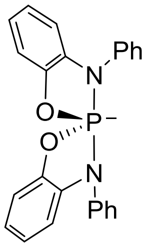

1ma⁻ :

N	-1.82533	0.65447	-0.47292
C	-1.87901	1.51967	0.58349
C	-0.57999	1.87810	0.98256
C	-2.95478	2.13253	1.22051
C	-0.34312	2.80437	1.96896
C	-2.71990	3.07748	2.21960
H	-3.96876	1.89936	0.92365
C	-1.43249	3.41214	2.59892
H	-1.26398	4.14512	3.37818
C	0.57971	-1.87765	0.98276
C	0.34265	-2.80421	1.96882
C	1.87882	-1.51948	0.58368
C	1.43186	-3.41253	2.59854
H	-0.67846	-3.04478	2.23648
C	2.95443	-2.13293	1.22043
C	2.71933	-3.07815	2.21923
H	3.56504	-3.55536	2.70102
O	0.40951	1.25605	0.30260
P	-0.00011	0.00027	-0.74762
H	-3.56572	3.55429	2.70160
H	0.67792	3.04515	2.23664
H	3.96847	-1.90005	0.92354
H	1.26316	-4.14574	3.37755
C	-2.89457	-0.09594	-0.93246
C	-3.89271	-0.60957	-0.09475
C	-2.95746	-0.41662	-2.29520
C	-4.91750	-1.38779	-0.60531
H	-3.83822	-0.41608	0.96813
C	-3.97579	-1.20893	-2.79445
H	-2.18744	-0.02914	-2.94969
C	-4.97159	-1.69474	-1.95750
H	-5.67566	-1.77249	0.06786
H	-3.99855	-1.43993	-3.85352
H	-5.77317	-2.30740	-2.35165

O	-0.40970	-1.25501	0.30321
N	1.82537	-0.65400	-0.47246
C	2.89481	0.09603	-0.93205
C	2.95749	0.41711	-2.29472
C	3.89345	0.60891	-0.09446
C	3.97604	1.20913	-2.79397
H	2.18715	0.03019	-2.94916
C	4.91843	1.38686	-0.60504
H	3.83919	0.41508	0.96837
C	4.97230	1.69423	-1.95715
H	3.99863	1.44042	-3.85298
H	5.67695	1.77100	0.06805
H	5.77402	2.30668	-2.35132

Sum of electronic and zero-point Energies= -1526.471328
Sum of electronic and thermal Energies= -1526.448576
Sum of electronic and thermal Enthalpies= -1526.447632
Sum of electronic and thermal Free Energies= -1526.525379

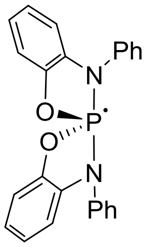

1ma• :

N	-2.04966	0.56166	-0.53507
C	-2.21378	1.67168	0.29261
C	-0.98286	2.28831	0.49564
C	-3.35907	2.21951	0.83955
C	-0.85075	3.44075	1.22923
C	-3.23802	3.38881	1.58684
H	-4.32374	1.75578	0.68338
C	-2.00595	3.99191	1.78158
H	-1.93568	4.90098	2.36407
C	0.68303	-1.69363	0.87549
C	0.36677	-2.84127	1.58060
C	2.02843	-1.43212	0.46921
C	1.35684	-3.76087	1.88299
H	-0.66143	-3.00046	1.87689
C	3.00934	-2.41216	0.77686
C	2.67538	-3.54868	1.46906
H	3.43199	-4.29292	1.68056
O	0.04320	1.64132	-0.14019
P	-0.38557	0.17608	-0.74631
H	-4.12578	3.83150	2.01961
H	0.12110	3.89583	1.36335
H	4.01943	-2.26665	0.42000
H	1.10137	-4.66077	2.42750
C	-3.06875	-0.38777	-0.79269
C	-3.76364	-0.98734	0.25312
C	-3.35110	-0.74934	-2.10379
C	-4.74243	-1.92880	-0.01578
H	-3.52470	-0.71401	1.27284
C	-4.31885	-1.70686	-2.36583
H	-2.81072	-0.27195	-2.91116
C	-5.02062	-2.29442	-1.32529
H	-5.28132	-2.38876	0.80297
H	-4.53114	-1.98462	-3.39040
H	-5.78180	-3.03540	-1.53290
O	-0.29312	-0.80775	0.62023
N	2.20148	-0.30663	-0.22426
C	3.42987	0.24153	-0.52142
C	3.57109	0.87518	-1.76290
C	4.50024	0.28793	0.38344
C	4.76213	1.48258	-2.10894
H	2.73012	0.86498	-2.44399
C	5.67842	0.92480	0.03723

H	4.38200	-0.13693	1.37145
C	5.82258	1.51126	-1.21186
H	4.86204	1.95135	-3.07973
H	6.48925	0.96860	0.75364
H	6.74943	2.00247	-1.47834

Sum of electronic and zero-point Energies= -1526.377557
Sum of electronic and thermal Energies= -1526.354758
Sum of electronic and thermal Enthalpies= -1526.353814
Sum of electronic and thermal Free Energies= -1526.433733

DFT calculations were performed using Gaussian 09.2.[10] Geometry optimizations of all molecules below (**1(MS)**$^{\mathbf{TBP}}$, **1(MS)**$^{\mathbf{SP}}$ and **1$^{\bullet}$(MS)**) were optimized using the uwB97XD/def2-TZVPP level of theory,[11,12] implemented in the Gaussian 09 software. CPCM was used to account for the effect of THF. Thermal energy corrections were extracted from the results of frequency analysis performed at the same level of theory. Frequency analysis of **1(MS)**$^{\mathbf{TBP}}$ and **1$^{\bullet}$(MS)** contained no imaginary frequency showing that these are energy minima, frequency analysis of **1(MS)**$^{\mathbf{SP}}$ contained one imaginary frequency showing that this is a TS related to the Berry pseudo-rotation.

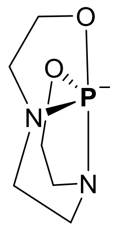

1(MS)$^{\mathbf{TBP}}$ (gas):

P	-0.17831	-0.47376	-0.84276
O	-1.85611	-1.08896	-0.55170
N	-0.75318	0.99527	-0.03907
C	-2.70178	-0.28331	0.18319
H	-3.26261	-0.85996	0.93662
H	-3.44784	0.20244	-0.47122
C	-1.85575	0.79167	0.85695
H	-2.42590	1.71735	1.00791
H	-1.50697	0.45673	1.85166
C	0.21722	1.96710	0.37374
H	-0.16789	2.98974	0.25600
H	0.47643	1.84859	1.44222
C	1.44682	1.72060	-0.50692
H	2.32595	2.24397	-0.10549
H	1.24849	2.13773	-1.50154
N	1.64011	0.30301	-0.61491
O	0.31046	-1.59768	0.34694
C	2.24835	-0.28233	0.54621
H	1.98333	0.21606	1.49819
H	3.34935	-0.30158	0.49331
C	1.68517	-1.69050	0.56969
H	1.85129	-2.19029	1.53034
H	2.16763	-2.28860	-0.21788

Sum of electronic and zero-point Energies= -837.052508
Sum of electronic and thermal Energies= -837.042497
Sum of electronic and thermal Enthalpies= -837.041553
Sum of electronic and thermal Free Energies= -837.088083

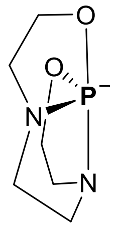

1(MS)^TBP (THF):

P	-0.13337	-0.48456	-0.78401
O	-1.77798	-1.25267	-0.39912
N	-0.79826	0.93367	-0.00346
C	-2.73130	-0.36774	0.08013
H	-3.42855	-0.86581	0.76738
H	-3.33535	0.06118	-0.73819
C	-1.98729	0.75684	0.78276
H	-2.58352	1.67500	0.81752
H	-1.74803	0.48352	1.82518
C	0.10670	1.97544	0.39764
H	-0.34866	2.96427	0.27345
H	0.38640	1.88336	1.46064
C	1.32431	1.79811	-0.50879
H	2.19354	2.34263	-0.12659
H	1.08859	2.21330	-1.49457
N	1.57580	0.38056	-0.62568
O	0.46100	-1.62406	0.33409
C	2.28057	-0.16051	0.51340
H	1.99677	0.30816	1.47099
H	3.36942	-0.06682	0.41775
C	1.85185	-1.61064	0.53380
H	2.07265	-2.10176	1.48414
H	2.36132	-2.16335	-0.26705

Sum of electronic and zero-point Energies= -837.128852
Sum of electronic and thermal Energies= -837.118868
Sum of electronic and thermal Enthalpies= -837.117924
Sum of electronic and thermal Free Energies= -837.164537

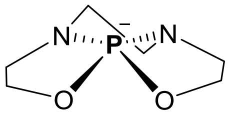

1(MS)^SP (gas):

P	-0.00672	-0.55767	-0.61214
O	-1.15462	-1.53726	0.32388
N	-1.22599	0.84292	-0.30911
C	-2.44285	-1.13061	0.04044
H	-3.14845	-1.62673	0.71632
H	-2.73225	-1.38260	-0.99539
C	-2.47366	0.38550	0.22073
H	-3.34920	0.83286	-0.27362
H	-2.55996	0.61524	1.29966
C	-0.68772	1.99056	0.35247
H	-1.24329	2.91033	0.11867
H	-0.71452	1.87602	1.45630
C	0.75502	2.09380	-0.11993
H	1.36605	2.62920	0.61937
H	0.79755	2.67643	-1.05421
N	1.21694	0.75526	-0.30279
O	1.13430	-1.58256	0.28447
C	2.44271	0.35812	0.30072
H	2.49118	0.62333	1.37524
H	3.32948	0.80086	-0.17916
C	2.43655	-1.15843	0.12699
H	3.08250	-1.65034	0.86438
H	2.82736	-1.40193	-0.87752

Sum of electronic and zero-point Energies= -837.044090
Sum of electronic and thermal Energies= -837.034767
Sum of electronic and thermal Enthalpies= -837.033823
Sum of electronic and thermal Free Energies= -837.078802

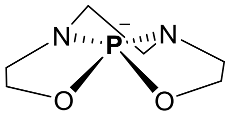

1(MS)^{SP} (THF):

P	-0.00842	-0.53736	-0.59811
O	-1.15664	-1.53221	0.33704
N	-1.21953	0.83999	-0.32133
C	-2.45273	-1.13106	0.03733
H	-3.16018	-1.61801	0.71319
H	-2.72972	-1.40418	-0.99414
C	-2.48265	0.38437	0.19514
H	-3.34492	0.82605	-0.31853
H	-2.58276	0.63056	1.26530
C	-0.67914	1.97697	0.37468
H	-1.24227	2.89413	0.16880
H	-0.70010	1.82709	1.47078
C	0.75552	2.09414	-0.10640
H	1.37502	2.61168	0.63353
H	0.79245	2.68919	-1.02984
N	1.21655	0.75089	-0.31872
O	1.13248	-1.58458	0.28459
C	2.44759	0.34887	0.28996
H	2.48721	0.61278	1.36122
H	3.32883	0.79621	-0.18624
C	2.44526	-1.16293	0.11579
H	3.09270	-1.65660	0.84592
H	2.82097	-1.41255	-0.89007

Sum of electronic and zero-point Energies= -837.120454
Sum of electronic and thermal Energies= -837.111170
Sum of electronic and thermal Enthalpies= -837.110226
Sum of electronic and thermal Free Energies= -837.155032

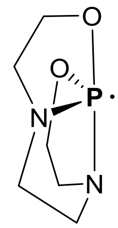

1$^{•}$(MS)^{TBP} (gas):

P	-0.08757	-0.48914	-0.48341
O	-1.49757	-1.41701	-0.34818
N	-0.86886	0.79275	0.26055
C	-2.64695	-0.62282	-0.18472
H	-3.41586	-1.22395	0.29958
H	-3.02607	-0.28891	-1.15726
C	-2.23382	0.58449	0.65966
H	-2.85138	1.45775	0.43622
H	-2.31596	0.37283	1.73201
C	-0.14554	2.02368	0.44034
H	-0.78118	2.88558	0.22434
H	0.22505	2.13178	1.46732
C	1.00191	1.90788	-0.57474
H	1.85630	2.52906	-0.30467
H	0.64634	2.23824	-1.55295
N	1.38317	0.51086	-0.64906
O	0.72420	-1.60643	0.37735

C	2.37230	0.07222	0.31459
H	2.22921	0.53799	1.30169
H	3.39008	0.28843	-0.01828
C	2.12846	-1.42135	0.41207
H	2.50339	-1.85002	1.33970
H	2.58218	-1.94425	-0.43349

Sum of electronic and zero-point Energies= -837.007087
Sum of electronic and thermal Energies= -836.997583
Sum of electronic and thermal Enthalpies= -836.996639
Sum of electronic and thermal Free Energies= -837.042501

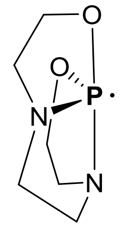

1•(MS)^{TBP} (THF):

P	-0.08629	-0.48264	-0.48152
O	-1.50236	-1.42226	-0.34751
N	-0.86399	0.78556	0.27069
C	-2.65207	-0.61502	-0.18959
H	-3.43113	-1.21070	0.28365
H	-3.01551	-0.27295	-1.16417
C	-2.23362	0.58093	0.66417
H	-2.84330	1.45917	0.44551
H	-2.31802	0.36180	1.73391
C	-0.14095	2.02140	0.44353
H	-0.78189	2.87846	0.23071
H	0.23578	2.12806	1.46681
C	0.99772	1.90902	-0.57953
H	1.85343	2.52888	-0.31393
H	0.63410	2.23979	-1.55419
N	1.38367	0.50833	-0.65939
O	0.72567	-1.61340	0.36836
C	2.36772	0.07395	0.31828
H	2.20909	0.54105	1.30040
H	3.38649	0.29663	-0.00355
C	2.13476	-1.41964	0.41509
H	2.50451	-1.84377	1.34601
H	2.59510	-1.94266	-0.42607

Sum of electronic and zero-point Energies= -837.015434
Sum of electronic and thermal Energies= -837.005928
Sum of electronic and thermal Enthalpies= -837.004984
Sum of electronic and thermal Free Energies= -837.050789

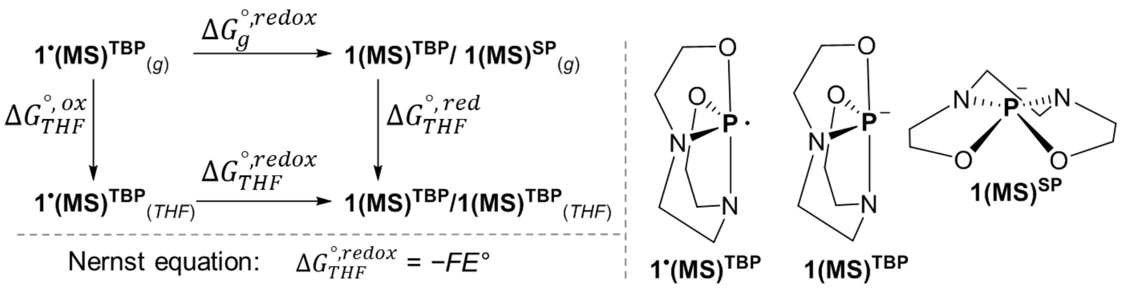

Figure S34. Thermodynamic Born−Haber cycle of **1(MS)^{TBP}** and of **1(MS)^{SP}**.

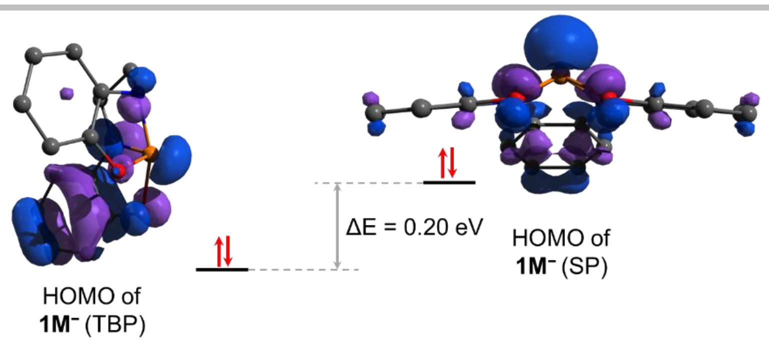

Figure S35. Energy difference between HOMO of **1m⁻** and **1ma⁻**.

EPR calculations of radicals 1•, [1-OCPh$_2$]• and [1-OC(C$_6$F$_5$)$_2$]•.

The EPR calculations for radicals **1•**, **[1-OCPh$_2$]•** and **[1-OC(C$_6$F$_5$)$_2$]•** were calculated at unrestricted TPSS0/def2-TZVPP level of theory[12] using the ORCA 4.0 software.[15] The calculated EPR parameters and spin density distributions are summarized in Tables 1 and 2, respectively.

Table S1. Experimental and DFT calculated EPR parameters for radicals **1•, [1-OCPh$_2$]•** and **[1-OC(C$_6$F$_5$)$_2$]•**.

	g_{iso}	a(^{31}P) [G]	a(^{14}N) [G]	a(^{19}F) [G]	a(^{1}H) [G]
**1•**$_{Exp.}$	2.003	607.2	10.2	-	-
**1•**$_{Calc.}$	2.003	596.7	10.3	-	-
**[1-OCPh$_2$]•**$_{Exp.}$	2.003	44.8	-	-	4H$_o$ = 3.3; 4H$_m$ = 1.3; 2H$_p$ = 3.6
**[1-OCPh$_2$]•**$_{Calc.}$	2.003	43.1	-	-	4H$_o$ = 4.4; 4H$_m$ = 2.3; 2H$_p$ = 4.5
**[1-OC(C$_6$F$_5$)$_2$]•**$_{Exp.}$	2.004	36.0	-	4F$_o$ = 5.8, 3.7, 4.6, 4.1; 4F$_m$ = 3.6, 3.6, 2.8, 2.7; 4F$_p$ = 7.5, 6.1	-
**[1-OC(C$_6$F$_5$)$_2$]•**$_{Calc.}$	2.003	37.7	-	4F$_o$ = 9.2, 5.7, 7.4, 6.6; 4F$_m$ = 5.9, 5.8, 4.5, 4.3; 4F$_p$ = 12.0, 9.7	-

7. References

[1] P. Chaudhuri, M. Hess, J. Müller, K. Hildenbrand, E. Bill, T. Weyhermüller, K. Wieghardt, *J. Am. Chem. Soc.,* **1999**, *121*, 9599-9610.

[2] Bruker (**2012**). Apex-II. Bruker AXS Inc., Madison, Wisconsin, USA.

[3] Bruker (**2013**). SAINT v8.34A. Bruker AXS Inc., Madison, Wisconsin, USA.

[4] Bruker (**2014/5**). Sadabs, 2014/5. Bruker AXS Inc., Madison, Wisconsin, USA.

[5] O. V. Dolomanov, L. J. Bourhis, R. J. Gildea, J. A. K. Howard, H. *J.* Puschmann, *Appl. Crystallogr*., **2009**, *42*, 339-341.

[6] G. M. Sheldrick, *Acta Cryst. A*, **2015**, *71*, 3-8.

[7] B. Tumanskii, O. Kalina, O. Radical Reactions of Fullerenes and their Derivatives, Kluwer Academic Publishers, Dordrecht, **2002**.

[8] A. Hirsch, M. Brettreich, Fullerenes: Chemistry and reactions, *Wiley-VCH*, Weinheim, **2005**.

[9] M. D. Tzirakis, M. Orfanopoulos, *Chem. Rev.* **2013**, *113*, 5262-5321.

[10] Gaussian 09, Revision D.01, M. J. Frisch, G. W. Trucks, H. B. Schlegel, G. E. Scuseria, M. A. Robb, J. R. Cheeseman, G. Scalmani, V. Barone, B. Mennucci, G. A. Petersson, H. Nakatsuji, M. Caricato, X. Li, H. P. Hratchian, A. F. Izmaylov, J. Bloino, G. Zheng, J. L. Sonnengerg, M. Hada, M. Ehara, K. Toyota, R. Fukuda, J. Hasegawa, M. Ishida, T. Nakajima, Y. Honda, O. Kitao, H. Nakai, T. Vreven, J. A. Montgomery Jr, J. E. Peralta, F. Ogliaro, M. Bearpark, J. J. Heyd, E. Brothers, K. N. Kudin, V. N. Staroverov, T. Keith, R. Kobayashi, J. Normand, K. Raghavachari, A. Rendell, J. C. Burant, S. S. Iyengar, J. Tomasi,

M. Cossi, N. Rega, N. J. Millam, M. Klene, J. E. Knox, J. B. Cross, V. Bakken, C. Adamo, J. Jaramillo, R. Gomperts, R. E. Stratmann, O. Yazyev, A. J. Austin, R. Cammi, C. Pomelli, J. W. Ochterski, R. L. Martin, K. Morokuma, V. G. Zakrzewski, G. A. Voth, P. Salvador, J. J. Dannenberg, S. Dapprich, A. D. Daniels, Ö. Farkas, J. B. Foresman, J. V. Ortiz, J. Cioslowski and D. J. Fox, Gaussian, Inc., Wallingford CT, **2013**.

[11] (a) F. Weigend, R. Ahlrichs, *Phys. Chem. Chem. Phys.* **2005**, 7, 3297-3305; (b) J.-D. Chai, M. Head-Gordon, *J. Chem. Phys.* **2008**, *128*, 084106; (c) J.-D. Chai, M. Head-Gordon, *Phys. Chem. Chem. Phys.* **2008**, *10*, 6615-6620; (d) S. Grimme, *J. Chem. Phys. A* **2005**, *109*, 3067-3077; (e) M. M. Quintal, A. Karton, M. A. Iron, A. D. Boese, J. M. L. Martin, *J. Chem. Phys. A* **2006**, *110*, 709-716.

[12] D. A. Pantazis, X.-Y. Chen, C. R. Landis, F. Neese, *J. Chem. Theory Comput.* **2008**, *4*, 908-919; b) A. Schäfer, C. Huber, R. Ahlrichs, *J. Chem. Phys.* **1994**, *100*, 5829-5835; c) A. Schäfer, H. Horn, R. Ahlrichs, *J. Chem. Phys.* **1992**, *97*, 2571-2577.

[13] (a) A.D. Becke, *Phys. Rev. A* **1988**, *38*, 3098-3100; (b) A.D. Becke, *J. Chem. Phys.* **1993**, *98*, 5648-5652.

[14] P. Schwerdtfeger, M. Dolg, W. H. E. Schwarz, G. A. Bowmaker, P. D. W. Boyd, *J. Chem. Phys.* **1989**, *91*, 1762-1774.

[15] The calculations were carried out using F. Neese, ORCA, version 4.0, an ab initio, density functional and semi-empirical program package (Max Planck Institute for Bioinorganic Chemistry: Mulheim an der Ruhr, Germany, **2012**).

[16] R. R. Fraser, T. S. Mansour, S. Savard, *J. Org. Chem.* **1985**, *50*, 3232-3234.

[17] J. J. Warren, T. A. Tronic, J. M. Mayer, *Chem. Rev.* **2010**, *110*, 6961-7001.

[18] N. G. Connelly, W. E. Geiger, *Chem. Rev.* **1996**, *96*, 877-910.

[19] C. F. Wise, R. G. Agarwal, J. M. Mayer, *J. Am. Chem. Soc.* **2020**, *142*, 10681-10691.
